# Secondary Ion Mass Spectrometry Imaging Reveals Changes in the Lipid Structure of the Plasma Membranes of Hippocampal Neurons following Drugs Affecting Neuronal Activity

**DOI:** 10.1021/acschemneuro.1c00031

**Published:** 2021-04-26

**Authors:** Paola Agüi-Gonzalez, Bao Guobin, Maria A. Gomes de Castro, Silvio O. Rizzoli, Nhu T. N. Phan

**Affiliations:** † Department of Neuro- and Sensory Physiology, University Medical Center Göttingen, Göttingen 37073, Germany; ‡ Center for Biostructural Imaging of Neurodegeneration, University Medical Center Göttingen, Göttingen 37075, Germany; § Department of Pharmacology and Toxicology, University Medical Center Göttingen, Göttingen 37075, Germany; ∥ Department of Chemistry and Molecular Biology, University of Gothenburg, Gothenburg 41296, Sweden

**Keywords:** ToF-SIMS, mass spectrometry imaging, lipids, neurons, membranes

## Abstract

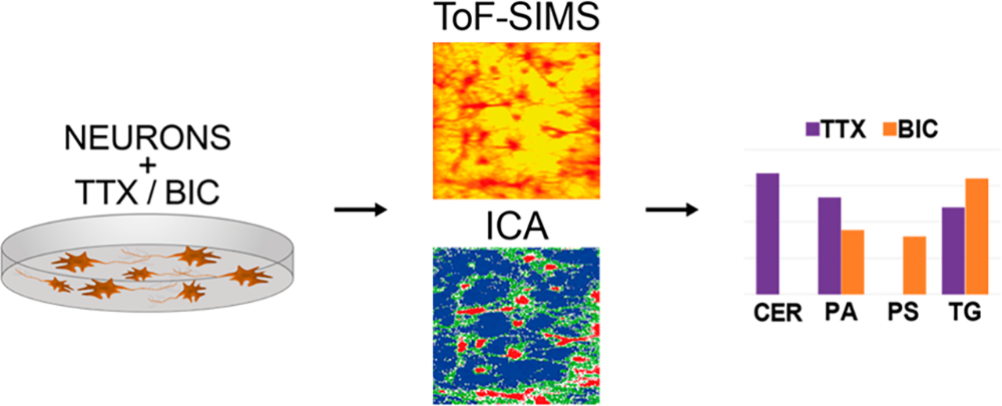

The cellular functions of lipids in the neuronal plasma membranes have been increasingly acknowledged, particularly their association to neuronal processes and synaptic plasticity. However, the knowledge of their regulatory mechanisms in neuronal cells remains sparse. To address this, we investigated the lipid organization of the plasma membranes of hippocampal neurons in relation to neuronal activity using secondary ion mass spectrometry imaging. The neurons were treated with drugs, particularly tetrodotoxin (TTX) and bicuculline (BIC), to induce chronic activation and silencing. Distinct lipid organization was found in the plasma membrane of the cell body and the neurites. Moreover, significant alterations of the levels of the membrane lipids, especially ceramides, phosphatidylserines, phosphatidic acids, and triacylglycerols, were observed under the TTX and BIC treatments. We suggest that many types of membrane lipids are affected by, and may be involved in, the regulation of neuronal function.

## Introduction

In the past decades, numerous studies have shown that lipids are not only the structural components but also highly dynamic biomolecules involved in neuronal processes, particularly in ion-channel regulation and synaptic plasticity.^1,2^ The cellular functions of lipids at neuronal plasma membranes have been increasingly interested in neuroscience and cell biology due to their association with physiological and pathological processes. Lipid diversity has been associated with the evolution of higher cognitive abilities, and it is affected by age, neuronal maturation, and stress levels.^3,4^ Alteration in lipid homeostasis has been proved to relate to neurodegenerative diseases, such as Alzheimer’s and Parkinson’s;^5,6^ neuropsychiatric afflictions, such as depression and bipolar disorder;^7−9^ and genetic diseases, such as Gaucher’s and Faber’s.^4,10^ Despite the evidence that these biomolecules are important in the functioning of cells and the brain, the current knowledge about the intact lipid organization of neuronal plasma membranes and their mechanistic regulation on neuronal activity remains sparse. This gap is mainly caused by a number of challenges, particularly the difficulties to preserve their intact structures for analysis and the lack of compatible labeling tools for imaging techniques.

Four major approaches have been available to visualize the lipid distribution on the cellular plasma membranes. The first approach is to use antibodies, for example PIP2 antibodies, to label specific lipid species. These affinity probes, however, exhibit several limitations for addressing the physiological distribution of lipids. In particular, the size of the probes limits the number of molecules that can be labeled within an area and that may cause a displacement of lipids. Also, protein-bound lipids might not be accessible for immunolabeling.^11^ In addition, antibodies are not permeable through the plasma membranes; thus, they are restricted to labeling the lipids at the outer layer of the membranes. The second approach utilizes fluorophore-conjugated lipids which enables live cell imaging. Despite their structural resemblance to the endogenous compounds, their different chemical properties alter the lipid arrangement and interaction with other membrane components.^12−14^ Third, rare isotopic lipids or lipid precursors are incorporated into the cellular membranes, which are subsequently detected by secondary ion mass spectrometry (SIMS). SIMS is a surface analysis technique that provides the chemical composition of samples by sputtering the surface with a highly focused primary ion beam. From the impacted region of the primary ion beam, the emitting secondary ions are extracted into the mass spectrometer, separated, and measured according to their mass to charge ratios (*m*/*z*). Elemental, isotopic, and molecular composition of the analyzed surface can be detected. Isotopic lipids have identical properties to those of the endogenous compounds, while they are distinguished by SIMS owing to their different *m*/*z* values. Nonetheless, the isotopic lipids are restricted to particular targets, and only a few number of lipid species can be detected simultaneously.^15,16^ Fourth, the membrane lipids are measured in an unlabeled fashion. Time-of-flight secondary ion mass spectrometry (ToF-SIMS) has been well-known as a powerful label-free imaging technique for simultaneous detection of various analytes within a mass range up to ∼1500 Da, including molecular lipids. ToF-SIMS provides a low detection limit (in the range ppm to ppb) and a relatively high spatial resolution (from ∼500 nm to a few μm).^17^ This approach has already been applied to study lipid distribution on tissues,^18^ single cells,^19^ and, more specifically, on neurons.^20,21^

Another limitation to understand the role of lipids in neuronal processes is that most of the available studies analyze large areas of the brain. Due to the heterogeneity of the tissue in different brain regions and considering, for example, the variance on the ratio of neurons per glia between subregions,^22^ it is difficult to extrapolate the abundance of different lipid species to the single neuron level.

In this paper, we investigated the lipid organization of the plasma membranes of hippocampal neurons and how it relates to the neuronal activity using ToF-SIMS imaging. Neuronal activity was modulated by treatments with drugs, particularly with tetrodotoxin (TTX) and bicuculline (BIC), to induce opposite effects and chronic changes in their synaptic activity.^23^ The differences in the lipid organizations between the neuronal cell body and neurites in different states of neuronal activity were analyzed using the multivariate independent component analysis method (ICA). It was found that the organization of membrane lipids is related to function, and several particular groups of lipids may be involved in regulating neuronal activity.

## Results and Discussion

### Sample Preparation of Hippocampal Neurons for SIMS Imaging

Following the protocol of Kaech and Banker,^24^ rat hippocampal neurons were cultured on indium tin oxide (ITO) coated glass slides to keep a physical distance with the astrocytic monolayer in a sandwich fashion, the so-called Banker cell culture. This protocol allows for the correct development of the neurons while reducing the interferences from other cell types and ensuring the analysis on neuronal plasma membranes. Moreover, a low density of neurons makes it easy to distinguish different cellular areas, particularly the cell body and neurites, both morphologically and biochemically^24^ allowing the study of the variation of lipid distribution in these regions.

**Figure 1:**
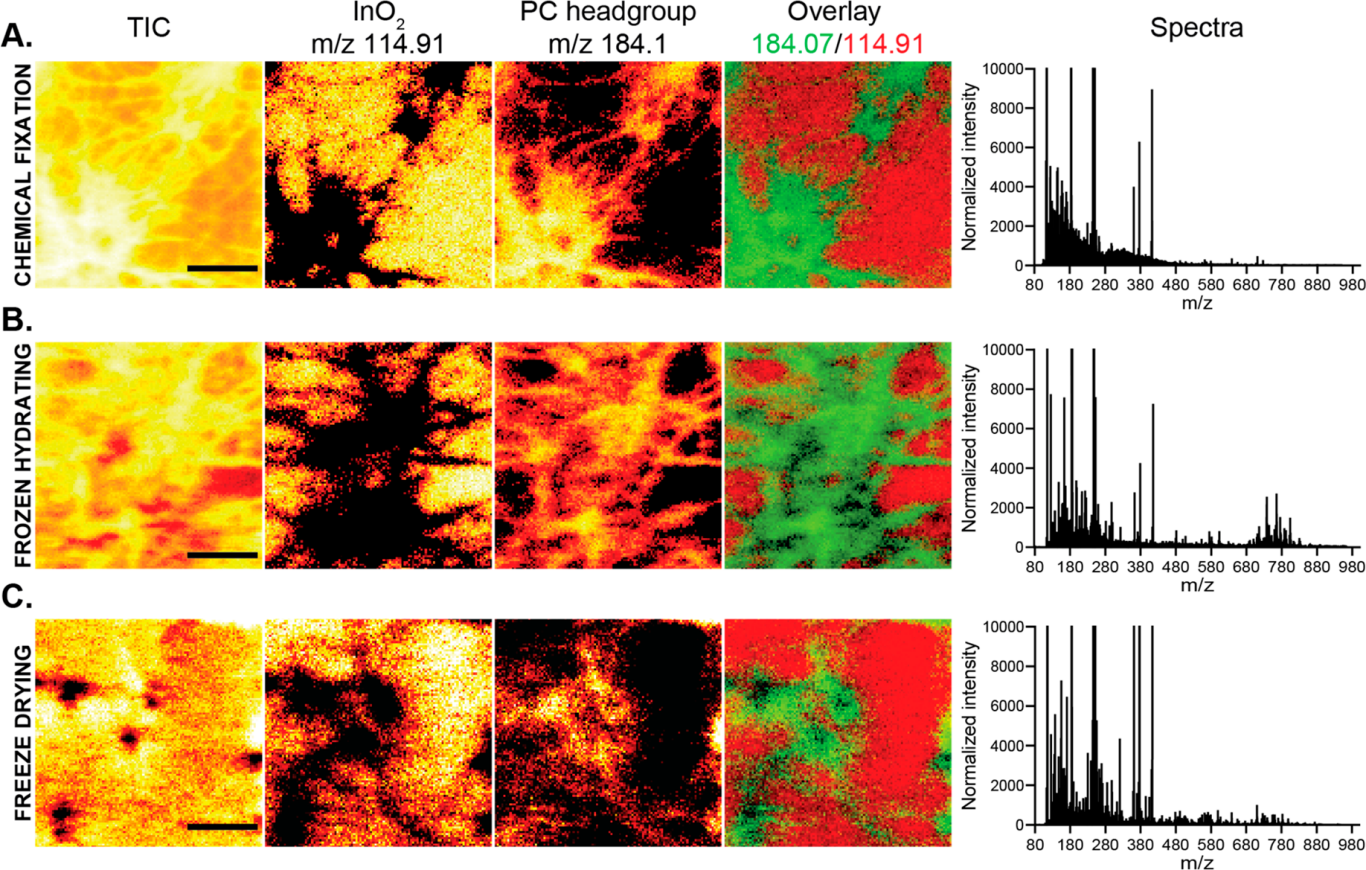
Comparison of sample preparation for hippocampal neurons using ToF-SIMS imaging. From left to right: Ion images of total ion count (TIC), InO_2_ at *m*/*z* 114.91, phosphatidylcholine (PC) headgroup at *m*/*z* 184.07, overlay of these ions, and spectra obtained from (A) chemically fixed, (B) frozen hydrated, and (C) freeze-dried samples. Scale bars: 100 μm.

The sample preparation methods, including chemical fixation, frozen hydrating, and freeze-drying, were examined regarding the cell morphology with preserved distribution of lipids and their signal intensity for ToF-SIMS measurements (Figure 1). It was shown that chemical fixation with 4% glutaraldehyde followed by 0.4% osmium tetroxide (OsO_4_) preserved the morphology of the cells well (Figure 1A). However, either lipid loss or extensive cross-linking of lipids in the membrane by chemical fixation possibly prevents the release of large fragment ions (above *m*/*z* 500), which appeared at very low intensities, and thus results in a loss of the lipid signatures of the cells. On the other hand, both frozen hydrating and freeze-drying methods preserved the cell morphology well with no apparent delocalization of the lipids and preserved the high mass signal better than that of the fixation (Figure 1B,C). Although frozen hydrated samples had higher high mass signals than those in the freeze-dried ones, the sample handling was greatly challenging leading to low reproducibility of the results. Freeze-drying has been commonly used to preserve biological samples for ToF-SIMS analysis due to the simplicity of the procedure and high reproducibility of the results. The method may cause some degree of shrinking and chemical rearrangement during dehydration; thus, great care must be taken to lower these risks. Freeze-drying, therefore, was chosen for our further experiments.

### Lipid Organization Across Neuronal Plasma Membranes Investigated by Independent Component Analysis (ICA)

We used independent component analysis (ICA) and neighborhood cross-correlation coefficient (NBCC) analysis to investigate the difference in lipid distribution between different regions of neuronal plasma membranes, particularly the cell body and neurites. We first selected at least three spectral data sets of the ion images which had well-recognized cell body and neurite areas. We then calculated the NBCC values for each pixel on individual spectral data sets. The NBCC values were found to be larger in the cell body than in the neurites, while the background had the smallest values. Afterward, we used a Gaussian mixture model (GMM) to classify the NBCC of each pixel into one of the three defined categories, namely, the cell body, neurites, and background. Based on the pixel probability maps, we created three masks that were more likely the cell body, neurites, and background.

We then grouped the spectral data of all pixels into three groups corresponding to the three masks and performed ICA. The independent components (ICs) contributing to the cell body, neurites, and background were then grouped into their categories. The number of ICs was determined by the principal component analysis (PCA) performed beforehand, and that covered more than 95% of the total data. Each IC is a group of different mass peaks which have a certain relationship to each other, for example, these peaks could be the compositional constituents of a protein or could be the fragments from a molecular lipid. Therefore, IC could contain the fingerprint of particular biomolecules in a very complex molecular mixture. These three IC-groups were subsequently used to unmix the rest of the data (*n* = 9), and their corresponding images were used to further refine the masks.

**Figure 2:**
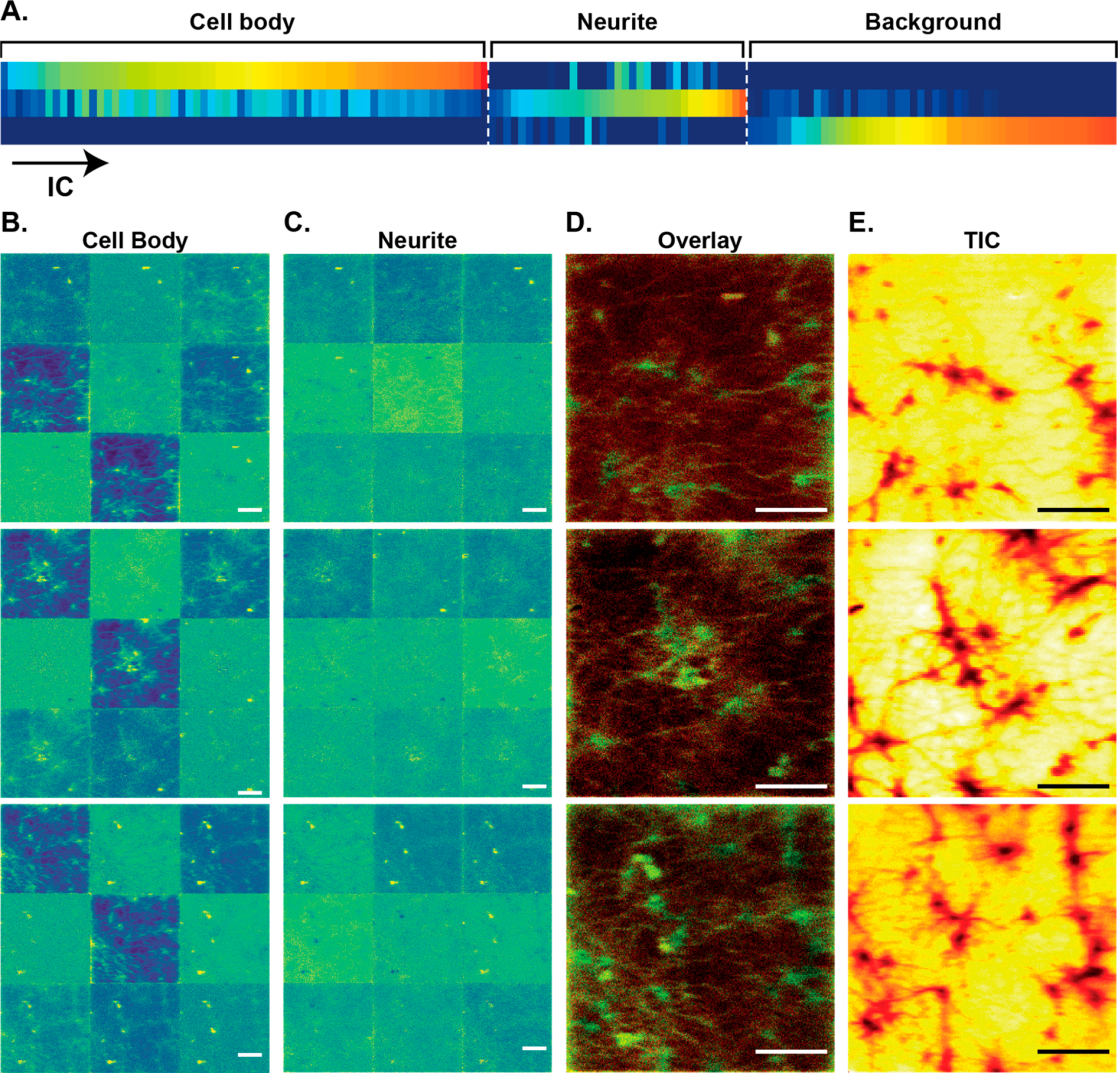
Different lipid distribution across neuronal plasma membranes shown by independent component analysis in positive ion mode. (A) Color map of ICs showing their contributions to the cell body, neurites, and background. Color scale represents the lowest (blue) to the highest (red) levels of contribution. IC-images of the first nine ICs dominating in the (B) cell body and the (C) neurites in three different samples (in three rows). (D) Overlay of the nine IC-images of the cell body (green) and neurites (red). (E) TIC images of the three samples. Scales bars: 200 μm.

The result showed that the molecular distribution was different between the cell body and the neurites. The ICs contributing to the cell body, neurites, and background were grouped into their categories (Figure 2A). The ICs mainly contributing to the cell body area (first line) are also present in the neurites, however at a significantly lower level (second line). Similarly, in the neurite areas, the main ICs of the neurites (second line) have a low contribution to the cell body (first line). These ICs are very distinct from those of the background (third line). It is noticed that there are a few ICs overlapping slightly between the neurites and background, which is explained by the neurites being so thin that it causes a low level of co-ionization and coextraction of the ITO substrate underneath the imaged neurite areas. The IC-images of the first nine ICs contributing to the cell body and the neurites for three different samples are shown in Figure 2, parts B and C, respectively (three rows). The IC-images of the cell body show part of the neurite area, whereas almost no cell body is observed in the IC-images of the neurites. The overlay images of the cell body and neurites (Figure 2D) show clear and continuous structures of the neurons and correlate well with the total ion images (Figure 2E).

Tracking the mass peak composition of individual ICs, we could obtain the information on lipid molecules and their fragments, which localize differently between the neuronal cell body and neurites. For example, for the second IC dominating in the cell body (IC index number 37) in positive mode, several peaks were found to be possibly related to each other, such as peaks at *m*/*z* 184.07, 224.10, 440.28, 478.35, and 649.62. The peaks C_5_H_15_NO_4_P at *m*/*z* 184.07 and C_8_H_19_NO_4_P at 224.10 have been commonly known as fragments of phosphatidylcholines (PCs), whereas the peaks at *m*/*z* 440.28 could be a fragment of PCs and phosphatidylethanolamine (PEs) having a specific fatty acid tail (C12:0, C15:0 in the PCs and PEs, respectively).^25^ The mass *m*/*z* 478.35 could be derived from PCs and PEs with fatty acid tails of C16:0 and C19:0, respectively. In addition, the peak at *m*/*z* 649.62 is possibly a diacylglycerol (DG), DG (39:0) (M–OH)^+^. Thus, the IC 37 contains signature fragments of a specific group of PCs and PEs which possibly have a certain structural and functional relationship at the membrane of the cell body. Another example is the third IC dominating in the neurites in positive mode (IC index number 3), where signature peaks were found at *m*/*z* 86.10 for the PC fragment, *m*/*z* 433.23 for phosphatidic acid PA ((15:0)+Na)^+^, *m*/*z* 577.54 for DG (34:1), and *m*/*z* 562.58 and *m*/*z* 660.64 for sphingomyelin SM (37:1) and SM (44:1) via neutral loss of 183. This IC shows an interesting relation between the fragments from different lipid groups. In negative mode, for the sixth IC dominating in the neurites (IC index number 6), possible related peaks are fatty acids (FAs), FA (14:0) at *m*/*z* 227.24, FA (16:0) at *m*/*z* 255.22, FA (18:2) at *m*/*z* 279.23, FA (18:1) at *m*/*z* 281.28, FA (18:0) at *m*/*z* 283.23, and FA (20:0) at *m*/*z* 311.30 and triacylglycerols (TGs), TG (50:0) at *m*/*z* 833.79 and TG (52:0) at *m*/*z* 861.81. These fatty acids could be the fragments of the TGs. The ICA therefore provides an advantage in the ability to elucidate the possible relationship of particular groups of lipids and their fragments. The signature fragments of the most dominant ICs in the cell body and neurites for both ion modes are listed in Table S1.

### Cross-Correlation Coefficient Difference (CCD) Analysis for Study of Neuronal Membrane Lipids following Drug Treatments

**Figure 3:**
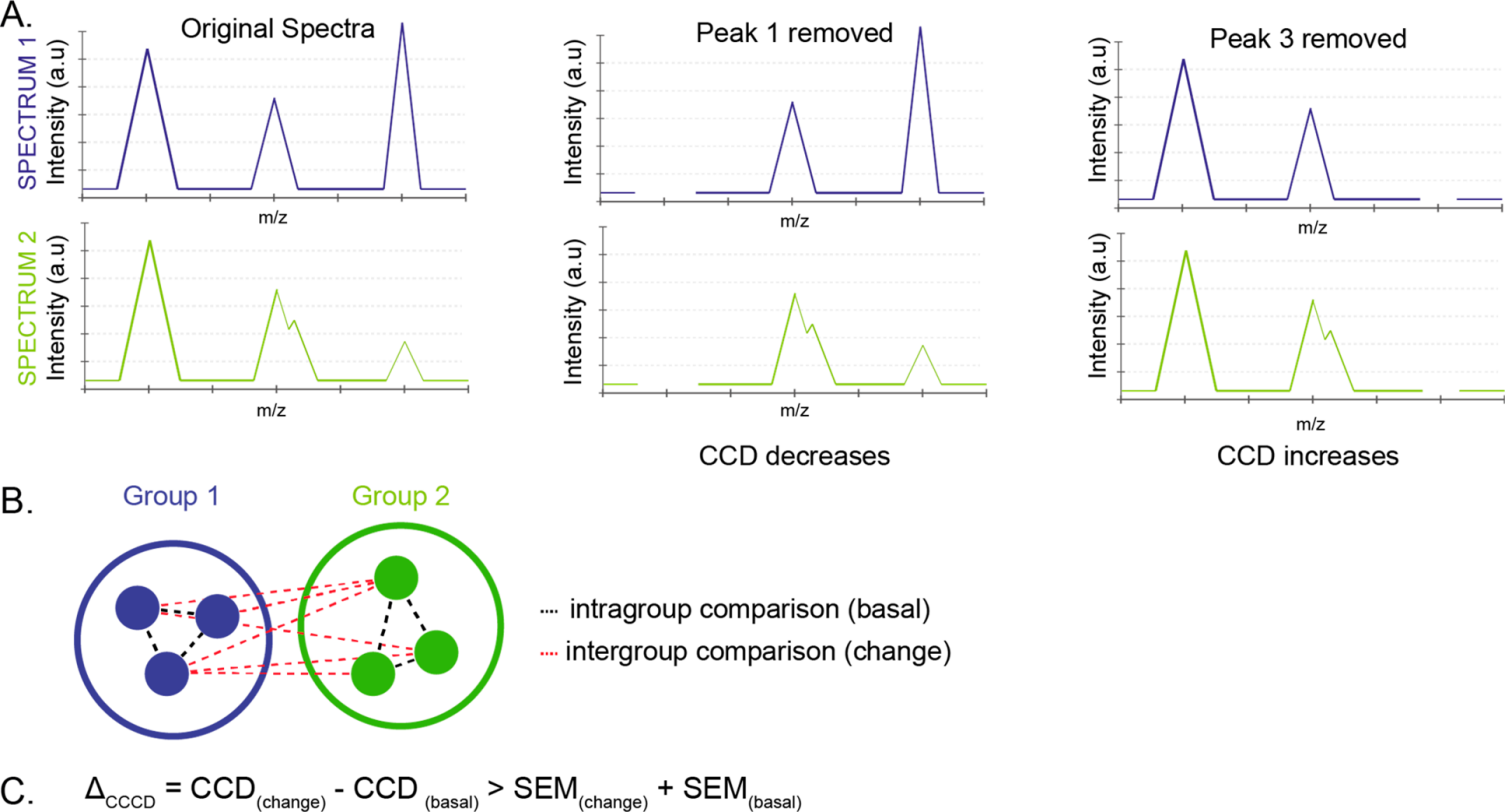
Cross-correlation coefficient differences analysis. (A) Comparison of a pair of spectra with and without a particular peak to determine the contribution of the peak to the spectral difference. CCD of the pair decreases if peak 1 is included but increases if peak 3 is included. (B) Comparison of CCD within the groups (basal) and between treatment and control groups (change) to determine which peaks significantly contribute to the difference between these groups based on (C) criteria.

To investigate how the lipid organization of neuronal plasma membranes changes following the drug treatments which alter the neuronal activity, we used cross-correlation coefficient difference (CCD) analysis. This method compares each pair of the spectra obtained before and after a peak of interest is removed in order to determine the contribution of that peak to the difference of these spectra. If the spectra look more similar after the peak is removed, it means that the peak contributes with a high score to the difference between these spectra, and thus, the CCD value of the spectra increases positively with that peak. On the contrary, if the spectra look more different after the peak is removed, the peak contributes with a low score, and the CCD value of the spectra decreases or increases negatively with that peak (Figure 3A). The larger value is the CCD value, and the higher value is the contribution of the peak to the difference of the spectra. We examined the molecular difference in the neuronal membranes between the drug-treated and the control groups by comparing the CCD of the spectra in pairwise for all the combinations between these groups (change) (Figure 3B). Similarly, we also compared the CCD of the spectra in pairwise within the control group (basal) (Figure 3B). Afterward, the mean of CCD of all the pairs and its standard deviations of the mean (SEM) were calculated for each mass peak. The peak was then considered as a significant peak contributing to the difference between the control and treatment groups based on the criteria that the mean of CCD of the change minus the mean of CCD of the basal was larger than their sum of SEM (Figure 3C), which was one sigma difference, and the confidence interval was larger than 0.683. The CCD analysis was carried out separately for the cell body and neurite areas of the neuronal membranes and in positive and negative SIMS modes.

The CCD analysis ensures a more reliable comparison than most of the common statistical tests, because it does not require any normalization of the data which often introduces a certain bias. In this manner, we can compare the contribution of each peak to the difference caused by the drug treatments while avoiding the distortion of the data due to the normalization process and, most importantly, maintaining the independence from the signal amplitudes by only comparing the shapes of spectra. In contrast to other methods such as the *t*-test, which only compares a single peak or a group of peaks between two spectra and neglects the rest of the spectra containing the majority of signals and noises, the CCD analysis compares two entire spectra in which only one target peak is missing; thus, it retains all the information to the greatest extent. In particular, it provides a balanced total noise level of the entire spectra, which are often not easy to normalize.

### Lipid Structural Changes in Neuronal Plasma Membranes under Drug Treatments

To examine the lipid structural changes in the neuronal plasma membranes following drug treatments using CCD analysis, we prepared several groups of hippocampal neurons which were treated with drugs to modulate neuronal activities. The first group was incubated with tetrodotoxin (TTX), a potent neurotoxin that binds to the voltage-gated sodium channels blocking the passage of sodium ions and inhibiting the firing of action potentials.^26^ The neurons therefore were induced to reduce their activity. The second group was treated with bicuculline (BIC), a competitive antagonist of GABA_A_ receptors, to enhance neuronal activity.^27^ The third group was the control which was prepared similarly but without adding a drug. To obtain the statistical results, three independent sets of neuronal samples, each set comprised of three conditions, control, TTX treatment, and BIC treatment, were prepared. ToF-SIMS imaging was then carried out on three or four different areas of each sample (*n* ≥ 27).

The CCD analysis was performed comparing the treatment and the control groups for cell body and neurite areas of the neuronal membranes in positive mode and negative mode. From the result, we identified 51 lipid-related peaks which significantly contribute to the difference between the TTX treatment and the control groups. On the other hand, from the comparison between BIC treatment and control groups, we identified 41 significant peaks assigned for lipids and lipid fragments. The lipid peaks significantly affected by the drug treatments are summarized in Table 1. Comparison of the CCD values between the treatments and control for the cell body and neurites are presented in Figures S1–S4 in positive ion mode and in Figures S5–S8 in negative ion mode. Different groups of lipids were found to significantly change when the neuronal activity was altered by the drug treatments. Particularly, in positive mode for TTX treatment, the PC fragments, including the peaks at *m*/*z* 166.06, 184.07, and 224.11 and ceramides with 34 and 36 carbon chains, caused the major difference in both the cell body and neurites. PAs, Phosphatidylglycerols (PGs), and phosphatidylserines (PSs), such as PA (12:0)+K, PG (28:0, O)+Na, and PS (43:6)+Na, also contributed to the difference but to a lesser extent. For BIC treatment, PCs and PAs changed the most followed by PGs and PSs. The change of PCs, however, mainly occurred in the cell body as shown by the changes of the PC fragments and several molecular species such as PC (30:0), PC (32:1)+Na, and PC (34:2)+Na. It was noticed that ceramide species were only affected by TTX treatment.

**Table 1:**
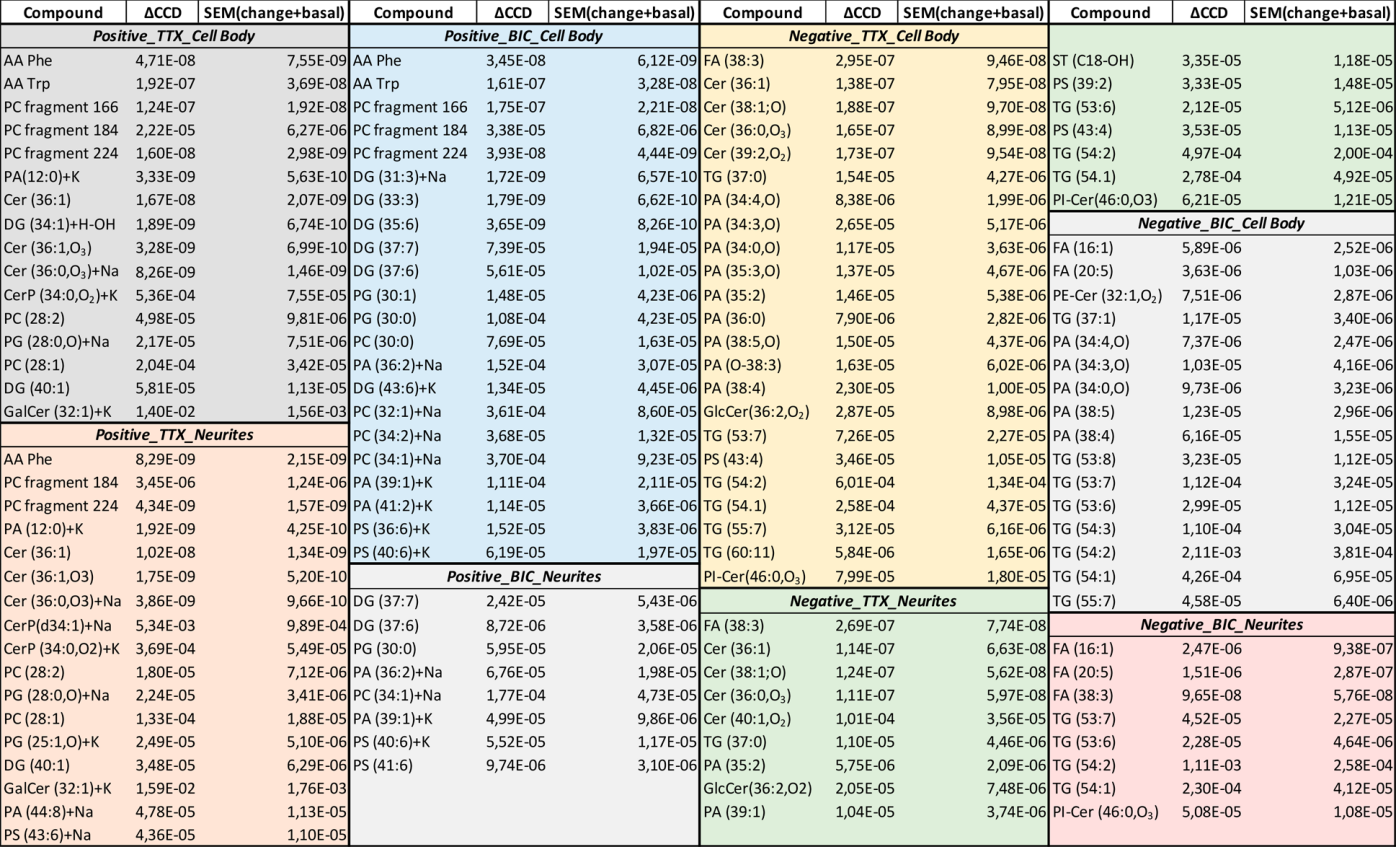
Summary of the Significant Lipid Peaks Affected by the Drug Treatments from the CCD Analysis

In negative mode for TTX treatment, the major changes were found in a number of ceramide (Cer), PA, and TG species in both the cell body and neurites. There were a larger number of PAs affected in the cell body compared to the neurites. On the other hand, the BIC treatment caused significant change in TGs in both the cell body and neurites, while altered PAs were mainly changed in the cell body. Comparison between TTX and BIC treatments showed that ceramides were mainly affected by the TTX, whereas FA (16:1) and FA (20:5) were only changed by the BIC.

The results showed that significant alteration in the lipid organization of the neuronal plasma membranes occurred following the drug treatments. Lipids and their fragments were affected differently by the two drugs, TTX and BIC, which induce opposite changes in the neuronal activity. This could be a significant correlation between the membrane lipid organization and the neuronal activity implying the involvement of membrane lipids in neuronal functioning.

### Lipid Organization of Neuronal Plasma Membranes and Its Link to Neuronal Plasticity

The long incubation period of neurons with TTX and BIC not only alters the immediate activity levels of the neurons but also induces homeostatic plasticity, promoting the adjustment of the overall strength of synapses.^23^ In our work, we showed that the change in the synaptic plasticity, which was induced by the TTX and BIC treatments, altered the lipid composition of the neuronal plasma membranes. This correlation suggests that lipids could be involved in an underlying molecular mechanism that contributes to the homeostatic plasticity in hippocampal neurons.

**Figure 4:**
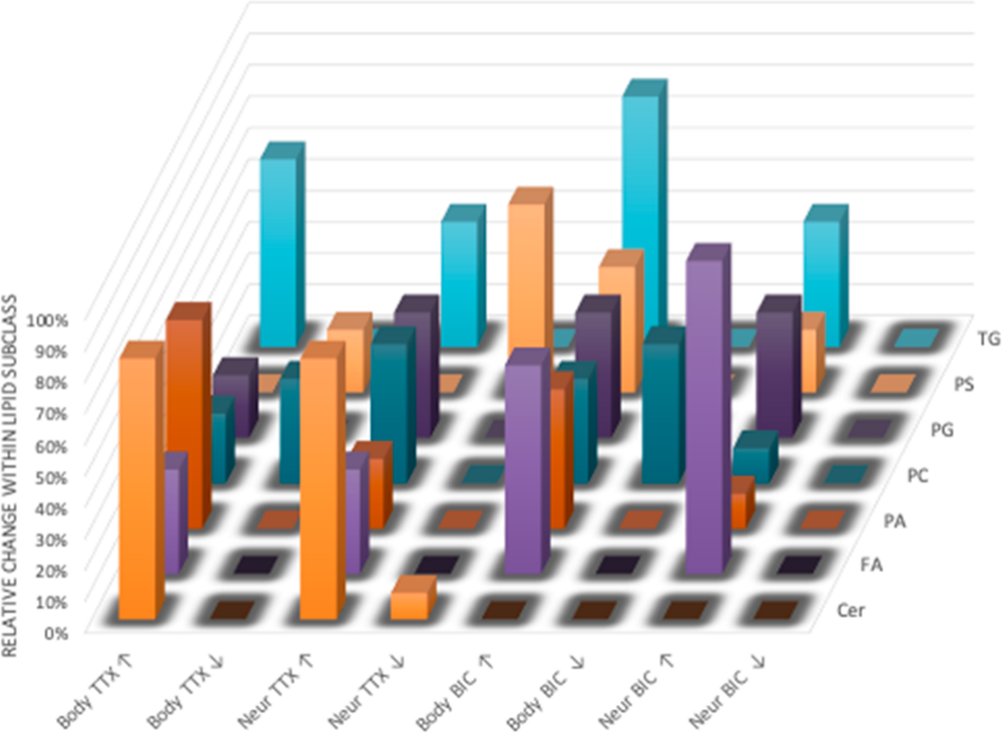
Alterations of the lipid compositions of the plasma membranes of hippocampal neurons following drug treatments (TTX, BIC). The trend of alteration within individual lipid subclasses was obtained by comparing their peak intensities from the treatments to those from the controls. The trends (increase as ↑ or decrease as ↓) in regions (cell body or neurites) under treatments (TTX or BIC) are presented in the *X* axis. Lipid subclasses are presented in the *Y* axis. The number of lipid compounds having a similar trend within their lipid subclass are expressed as percentages in the *Z* axis.

To obtain an overview of how the membrane lipid structure changes corresponding to the neuronal activity, we examined the relative changes in the abundance of lipids within specific lipid groups after the neurons were treated with TTX or BIC. The compositional changes within the cell body and neurites were also analyzed. We sorted all tentatively assigned peaks of lipids into different subclasses, each of which contained at least three assigned peaks (*n* ≥ 3) (Table S2). The subclasses included in the analysis were Cer (*n* = 12), FAs (*n* = 3), PAs (*n* = 18), PCs (*n* = 9), PGs (*n* = 5), PSs (*n* = 5), and TGs (*n* = 11). Other lipids such as PEs or PIs were not included because of their insufficient number of assigned peaks. The average intensity of each peak was compared between the treatments and the control. Afterward, we calculated the number of lipids as the percentage within each subclass that changes in a similar trend (increase or decrease in abundance compared to the control). The overall trends of alteration in the lipid abundance within individual subclasses following the drug treatments are summarized in Figure 4 and Table S3.

From the analysis, we obtained a distribution of alteration that is certainly different from what would be expected from a random one, supporting that the lipid composition of neuronal plasma membranes is clearly influenced by the change of synaptic activity. It was clearly observed that different lipid subclasses were affected differently by the treatments. In addition, the membrane lipid composition of the cell body and the neurites also changed with different patterns. Several significant features were noticed. First, 83% of ceramides were considerably increased in their abundance in both the cell body and neurite areas when the neuronal activity was silenced by TTX; however, none of this lipid species was affected when the neuronal activity was activated by BIC. Second, the overall amount of PCs was decreased in the cell body (approximately 10% of PCs); however, it was increased in the neurites under the two treatments (44% with TTX, 11% with BIC). Third, PSs exhibited a dramatic reduction in the abundance in all the cells by the TTX treatment (20% the cell body, 60% the neurites). On the contrary, an increasing trend was observed for these lipid species with the BIC treatment (40% in cell body, 20% in neurites). In addition, the amounts of PAs, PGs, and fatty acids were elevated in both areas by the two drugs. Likewise, a dramatic elevation in the amount of TGs was observed in both regions by both treatments; noticeably, 60 and 80% of TGs in the cell body were enhanced by the TTX and BIC treatments, respectively.

Furthermore, to observe if there is any significant correlation between a particular group of lipids with the neuronal activity in the two cellular regions, we checked the significance of the data using a Kruskal–Wallis test combined with a Tukey-kramer posthoc and a correction parameter, α at 0.05. Although the levels of confidence were not sufficient to confirm all the trends due to the low number of assigned lipid peaks in a few subclasses, several lipid groups had clear, statistically distinct behaviors. Particularly, ceramides were shown to significantly increase their abundance in both the cell body and neurites by the TTX treatment, compared to the BIC. In addition, the concentration of PAs was elevated in both the cell body and neurites by the two treatments; however, it was significantly higher in the cell body than that in the neurites, remarkably by the TTX (45% difference).

Ceramides are among the major secondary messengers in the brain as well as the main components in the metabolic pathways of sphingolipids, one of the major classes of lipids in the plasma membranes. Ceramides play an important role in changing the spatial organization of cellular membranes, activating target proteins and clustering signaling molecules for cellular processes. Ceramides present in the cell membranes at a low concentration (≤4 mol %),^28^ however, efficiently influence the structure and dynamics of the membranes.^4,29,30^ Interestingly, our results show that the suppression of neuronal activity by the TTX treatment dramatically increases the membrane ceramides to adapt to the firing silencing. The activation of neuronal activity by the BIC, however, does not induce the accumulation of this lipid species.

Previous studies showed that ceramides were involved in modulating the synaptic activation.^31,32^ An increase of the ceramide amount in capsaicin-sensitive adult and embryonic sensory neurons by an incubation with exogenous ceramides or by a production of endogenous ceramides via the activation of sphingomyelinase were shown to significantly increase the number of action potentials.^33^ In addition, a pro-inflammatory agent, nerve growth factor (NGF), which is elevated during the inflammation in the sensory neurons, was shown to increase the number of action potentials. The NGF was also known to trigger the activation of the sphingomyelin signaling pathway, to release ceramides.^34^ Another study showed that synaptic excitation could be conducted along the nerve fiber without action potentials but via a production of ceramides in the membrane lipid rafts, which increased the intracellular calcium concentration and triggered the release of other secondary messengers including nitric oxide and cyclic guanosin monophosphate (cGMP).^35^ In our study, the firing silencing in hippocampal neurons by TTX induces an elevation of ceramides possibly via an activation of the sphingomyelin signaling pathway. This could result in the increased concentrations of intracellular calcium, nitric oxide, and cGMP, which subsequently mediate the conduction of synaptic activation in the absence of firing caused by TTX. Under the BIC treatment, the synaptic activity is already established; thus, the ceramide pathway does not change.

PSs are the main anionic phospholipids in the neuronal plasma membranes. PSs have been shown to be involved in several important signaling pathways related to membrane fusion and exocytosis.^36−38^ In addition, the lipids affect the metabolism and release of neurotransmitters such as dopamine, acetylcholine, norepinephrine, and serotonin.^39^ A decrease in PS level and the major constitution docosahexaenoic acid (DHA) were found to correlate well with cognitive impairment and Alzheimer’s disease.^40^ On the other hand, a dietary supplement of PS was shown to improve learning ability and short-term memory in animals and humans.^41,42^ This positive effect has been claimed due to the incorporation of the supplement PS into the neuronal membranes which helps to mediate the synaptic activity and to enhance synaptic plasticity.^42,43^ Our results show that the neuronal activity suppressed by TTX causes a reduction in the levels of membrane PS, whereas the activity activated by BIC enhances membrane PSs.

PSs have a strong interaction via an electrostatic force with GABA,^44^ an inhibitory neurotransmitter functioning to maintain the inhibition that counterbalances neuronal excitation. Among different phospholipids, only PSs were able to stimulate the GABA uptake into the synaptic membrane of rat and rabbit brain synaptosomes.^45,46^ This means that the amount of PSs in the synaptic membrane probably changes according to a need of the GABA uptake. Therefore, in the suppression state of neuronal activity by TTX, a need for the GABA uptake into synaptic membrane is low, resulting in a reduced amount of PS in the membrane. On the contrary, the BIC treatment blocking GABA receptors to increase neuronal activity possibly stimulates the GABA uptake for the excitation counterbalance, which then triggers the elevation of PS in the membrane. This could implicate an essential role of PSs in regulating the neuronal activity by adjusting their abundance in the plasma membranes.

Moreover, PAs and TGs are both commonly known as the precursors of glycerophospholipids and phospholipids, respectively.^47,48^ PAs have also been known to be involved in changing the membrane organization^49^ and mediating the penetration of proteins into the cellular membrane.^50^ From our study, the PA and TG levels are increased significantly by the two drug treatments, possibly to support the enhancement of different phospholipids in the membranes as the response to the change of neuronal activity.

In summary, the lipid organization of neuronal plasma membranes reflects the status of the neuronal activity. Significant alterations of the lipid levels, particularly ceramides, PSs, PAs, and TGs, were observed under the TTX and BIC treatments. This correlation suggests that membrane lipids change their organization to fit to, or even to mediate, neuronal and synaptic activity.

## Materials and Methods

### Cell Culture

Hippocampal neurons were obtained from dissociated hippocampi of embryonic rats (E18).^51^ Following the protocol of Kaech & Banker,^24^ the neurons were plated on indium tin oxide (ITO) glass slides which were previously coated with 1 mg/mL poly-l-lysine. The cells were cultured in 5% CO_2_ atmosphere at 37 °C for 14 days in the N2 medium and then divided into three different groups. The first and second groups were treated with tetrodotoxin (TTX) at 1.5 μM and bicuculline (BIC) at 1.2 μM, respectively, in N2 medium for 72 h. The third group, the control, was treated without drugs in the same manner to the drug-treated groups. Afterward, the cells were prepared following one of the procedures, namely, chemical fixation, frozen hydrating, and freeze-drying.

### Chemical Fixation

The chemical fixation was carried out following the procedure published elsewhere.^52^ In brief, the cells were rinsed with Hendry phosphate buffer (HPB) pH 7.4 and fixed with glutaraldehyde 4% in HPB at room temperature for 30 min. The fixed cells were sequentially washed with HPS two times, each for 5 min, and with triple distilled water. Afterward, the cells were fixed with osmium tetroxide (OsO_4_) 0.4% at room temperature for 15 min followed by washing with triple distilled water three times, each for 5 min. The samples were then allowed to air-dry before ToF-SIMS measurement.

### Frozen Hydrating

The cells were washed with PBS for 5 min and quickly rinsed four times with ammonium formate 150 mM at pH 7.4. Immediately after rinsing, the cells were quickly frozen in liquid propane. The samples were then stored in liquid nitrogen until they were transferred to the precooled ToF-SIMS instrument for measurement.

### Freeze-Drying

The cells were washed with PBS for 5 min and quickly rinsed four times with ammonium formate 150 mM at pH 7.4. Immediately after rinsing, the cells were quickly frozen in liquid propane followed by freeze-drying overnight in a freeze-dryer (Christ 2-4 LDPlus, Christ Martin, Germany) at a pressure of 0.05 mbar. The samples were stored for a short time in a vacuum chamber until they were transferred into the ToF-SIMS instrument for measurement.

### ToF-SIMS Imaging

ToF-SIMS imaging was carried out by a J105 3D Chemical Analyzer (Ionoptika Ltd., UK) using a 40 keV (CO_2_)_2500_^+^ gas cluster ion beam (GCIB) as the primary ion beam. The focus of the ion beam was adjusted using different apertures. The imaging was acquired in static mode in both positive and negative ion modes. The primary ion current of 12 pA was used to raster the sample areas of 750 μm to obtain images of 256 pixels × 256 pixels, which resulted in the primary ion dose density of 2 × 10^13^ ion/cm^2^. The detected mass range was set from *m*/*z* 80 to 1000 Da. For the frozen hydrated samples, the preparation chamber and analysis chamber of the J105 were cooled down to ≤200 and ≤90 K, respectively, before the samples were inserted. The temperature of the analysis chamber was maintained at ≤90K during the measurement. On the other hand, the analysis of the freeze-dried samples was performed at room temperature. To remove the potentially occurring ice layer on the surface of the frozen hydrated samples or possible surface contamination on freeze-dried samples, the first layer of the samples was eroded using the primary ion current of 4 × 10^13^ ion/cm^2^ on an area of 800 μm with 128 pixels × 128 pixels.

### Data Analysis

The data obtained from the J105 were exported and analyzed using Ionoptika Image Analyzer software (Ionopika Ltd., Southampton, UK). Further processing on image data was carried out using Matlab (The Mathworks, Inc., version R2017a). All the spectra extracted from the image data were binned down to 0.05 Da which resulted in the mass accuracy of around 62 parts per million (ppm) at *m*/*z* 800. The spectra were then filtered from the peaks of the ITO substrate and normalized to the total ion counts. The spectral data were further processed using independent component analysis (ICA) and cross-correlation coefficient difference (CCD) analysis to examine the differences in the lipid structure of the neuronal plasma membranes in different cellular regions and to study how these lipid structure changes following the drug treatments. Afterward, the masses significantly contributing to the difference between the treatment and control groups were assigned based on the literature and databases such as the Lipid Maps^53^ and LipidBlast.^25^ Finally, the Kruskal–Wallis test combined with the Tukey-kramer posthoc test was performed to examine the correlation between the changes of different lipid groups and the neuronal activity. The test was carried out with the correction α of 0.05 on individual groups of lipids.

## Supporting Information Available

Independent components distributing at plasma membrane of cell body and neurites obtained by ICA, assigned peaks in positive and negative SIMS mode, relative change of membrane lipid compositions in hippocampal neurons following drug treatments, and figures for comparison of CCD values between control and TTX treatment and between control and BIC treatment in the cell body and the neurites in positive and negative ion modes (PDF)

## References

[ref1] Rohrbough J.; Broadie K. (2005) Lipid Regulation of the Synaptic Vesicle Cycle. Nat. Rev. Neurosci. 6 (2), 139–150. 10.1038/nrn1608. 15685219

[ref2] Puchkov D.; Haucke V. (2013) Greasing the Synaptic Vesicle Cycle by Membrane Lipids. Trends Cell Biol. 23 (10), 493–503. 10.1016/j.tcb.2013.05.002. 23756446

[ref3] Lauwers E.; Goodchild R.; Verstreken P. (2016) Membrane Lipids in Presynaptic Function and Disease. Neuron 90 (1), 11–25. 10.1016/j.neuron.2016.02.033. 27054615

[ref4] Mencarelli C.; Martinez-Martinez P. (2013) Ceramide Function in the Brain: When a Slight Tilt Is Enough. Cell. Mol. Life Sci. 70 (2), 181–203. 10.1007/s00018-012-1038-x. 22729185 PMC3535405

[ref5] Kao Y. C.; Ho P. C.; Tu Y. K.; Jou I. M.; Tsai K. J. (2020) Lipids and Alzheimer’s Disease. Int. J. Mol. Sci. 21 (4), 1–37. 10.3390/ijms21041505. PMC7073164 32098382

[ref6] Gónzalez de San Román E.; Manuel I.; Giralt M. T.; Ferrer I.; Rodríguez-Puertas R. (2017) Imaging Mass Spectrometry (IMS) of Cortical Lipids from Preclinical to Severe Stages of Alzheimer’s Disease. Biochim. Biophys. Acta, Biomembr. 1859 (9), 1604–1614. 10.1016/j.bbamem.2017.05.009. 28527668

[ref7] Merrill C. B.; Basit A.; Armirotti A.; Jia Y.; Gall C. M.; Lynch G.; Piomelli D. (2017) Patch Clamp-Assisted Single Neuron Lipidomics. Sci. Rep. 7 (1), 1–8. 10.1038/s41598-017-05607-3. 28706218 PMC5509708

[ref8] Bozzatello P.; Brignolo E.; De Grandi E.; Bellino S. (2016) Supplementation with Omega-3 Fatty Acids in Psychiatric Disorders: A Review of Literature Data. J. Clin. Med. 5 (8), 67 10.3390/jcm5080067. PMC4999787 27472373

[ref9] Frajerman A.; Kebir O.; Chaumette B.; Tessier C.; Lamazière A.; Nuss P.; Krebs M.-O. (2020) Lipides Membranaires Dans La Schizophrénie et La Psychose Débutante: De Potentiels Biomarqueurs et Pistes Thérapeutiques?. Encephale 46 (3), 209–216. 10.1016/j.encep.2019.11.009. 32151446

[ref10] Alessenko A. V.; Albi E. (2020) Exploring Sphingolipid Implications in Neurodegeneration. Front. Neurol. 11 (5), 1–13. 10.3389/fneur.2020.00437. 32528400 PMC7254877

[ref11] Gorman B. L.; Kraft M. L. (2020) High-Resolution Secondary Ion Mass Spectrometry Analysis of Cell Membranes. Anal. Chem. 92 (2), 1645–1652. 10.1021/acs.analchem.9b04492. 31854976

[ref12] Wilson R. L.; Frisz J. F.; Hanafin W. P.; Carpenter K. J.; Hutcheon I. D.; Weber P. K.; Kraft M. L. (2012) Fluorinated Colloidal Gold Immunolabels for Imaging Select Proteins in Parallel with Lipids Using High-Resolution Secondary Ion Mass Spectrometry. Bioconjugate Chem. 23 (3), 450–460. 10.1021/bc200482z. PMC3951754 22284327

[ref13] Kraft M. L. (2017) Sphingolipid Organization in the Plasma Membrane and the Mechanisms That Influence. Front. Cell Dev. Biol. 4, 154 10.3389/fcell.2016.00154. 28119913 PMC5222807

[ref14] Kim R.; Lou K.; Kraft M. L. (2013) A New, Long-Wavelength Borondipyrromethene Sphingosine for Studying Sphingolipid Dynamics in Live Cells. J. Lipid Res. 54 (1), 265–275. 10.1194/jlr.D029207. 23129779 PMC3520533

[ref15] Yeager A. N.; Weber P. K.; Kraft M. L. (2016) Three-Dimensional Imaging of Cholesterol and Sphingolipids within a Madin-Darby Canine Kidney Cell. Biointerphases 11 (2), 02A309 10.1116/1.4939681. 26746168

[ref16] He C.; Weston T. A.; Jung R. S.; Heizer P.; Larsson M.; Hu X.; Allan C. M.; Tontonoz P.; Reue K.; Beigneux A. P.; et al. (2018) NanoSIMS Analysis of Intravascular Lipolysis and Lipid Movement across Capillaries and into Cardiomyocytes. Cell Metab. 27 (5), 1055–1066. e3. 10.1016/j.cmet.2018.03.017. 29719224 PMC5945212

[ref17] Agüi-Gonzalez P.; Jähne S.; Phan N. T. N. (2019) SIMS Imaging in Neurobiology and Cell Biology. J. Anal. At. Spectrom. 34 (7), 1355–1368. 10.1039/C9JA00118B.

[ref18] Philipsen M. H.; Phan N. T. N.; Fletcher J. S.; Malmberg P.; Ewing A. G. (2018) Mass Spectrometry Imaging Shows Cocaine and Methylphenidate Have Opposite Effects on Major Lipids in Drosophila Brain. ACS Chem. Neurosci. 9 (6), 1462–1468. 10.1021/acschemneuro.8b00046. 29508991

[ref19] Ren L.; Dowlatshahi Pour M.; Malmberg P.; Ewing A. G. (2019) Altered Lipid Composition of Secretory Cells Following Exposure to Zinc Can Be Correlated to Changes in Exocytosis. Chem. - Eur. J. 25 (21), 5406–5411. 10.1002/chem.201900010. 30762272

[ref20] Van Nuffel S.; Parmenter C.; Scurr D. J.; Russell N. A.; Zelzer M. (2016) Multivariate Analysis of 3D ToF-SIMS Images: Method Validation and Application to Cultured Neuronal Networks. Analyst 141 (1), 90–95. 10.1039/C5AN01743B. 26609549

[ref21] Passarelli M. K.; Ewing A. G.; Winograd N. (2013) Single-Cell Lipidomics: Characterizing and Imaging Lipids on the Surface of Individual Aplysia Californica Neurons with Cluster Secondary Ion Mass Spectrometry. Anal. Chem. 85 (4), 2231–2238. 10.1021/ac303038j. 23323749 PMC3867296

[ref22] Christensen J. R.; Larsen K. B.; Lisanby S. H.; Scalia J.; Arango V.; Dwork A. J.; Pakkenberg B. (2007) Neocortical and Hippocampal Neuron and Glial Cell Numbers in the Rhesus Monkey. Anat. Rec. 290 (3), 330–340. 10.1002/ar.20504. 17525948

[ref23] Turrigiano G. (2012) Homeostatic Synaptic Plasticity: Local and Global Mechanisms for Stabilizing Neuronal Function. Cold Spring Harb. Perspect. Biol. 4 (1), 1–18. 10.1101/cshperspect.a005736. PMC3249629 22086977

[ref24] Kaech S.; Banker G. (2006) Culturing Hippocampal. Nat. Protoc. 1 (5), 2406–2415. 10.1038/nprot.2006.356. 17406484

[ref25] Kind T.; Liu K. H.; Lee D. Y.; Defelice B.; Meissen J. K.; Fiehn O. (2013) LipidBlast in Silico Tandem Mass Spectrometry Database for Lipid Identification. Nat. Methods 10 (8), 755–758. 10.1038/nmeth.2551. 23817071 PMC3731409

[ref26] Lago J.; Rodríguez L. P.; Blanco L.; Vieites J. M.; Cabado A. G. (2015) Tetrodotoxin, an Extremely Potent Marine Neurotoxin: Distribution, Toxicity, Origin and Therapeutical Uses. Mar. Drugs 13 (10), 6384–6406. 10.3390/md13106384. 26492253 PMC4626696

[ref27] Johnston G. A. R. (2013) Advantages of an Antagonist: Bicuculline and Other GABA Antagonists. Br. J. Pharmacol. 169 (2), 328–336. 10.1111/bph.12127. 23425285 PMC3651659

[ref28] Silva L. C.; De Almeida R. F. M.; Castro B. M.; Fedorov A.; Prieto M. (2007) Ceramide-Domain Formation and Collapse in Lipid Rafts: Membrane Reorganization by an Apoptotic Lipid. Biophys. J. 92 (2), 502–516. 10.1529/biophysj.106.091876. 17056734 PMC1751408

[ref29] Van Blitterswijk W. J.; Van Der Luit A. H.; Veldman R. J.; Verheij M.; Borst J. (2003) Ceramide: Second Messenger or Modulator of Membrane Structure and Dynamics?. Biochem. J. 369 (2), 199–211. 10.1042/BJ20021528. 12408751 PMC1223095

[ref30] Cremesti A. E.; Goni F. M.; Kolesnick R. (2002) Role of Sphingomyelinase and Ceramide in Modulating Rafts: Do Biophysical Properties Determine Biologic Outcome?. FEBS Lett. 531 (1), 47–53. 10.1016/S0014-5793(02)03489-0. 12401201

[ref31] Yang S. N. (2000) Ceramide-Induced Sustained Depression of Synaptic Currents Mediated by Ionotropic Glutamate Receptors in the Hippocampus: An Essential Role of Postsynaptic Protein Phosphatases. Neuroscience 96 (2), 253–258. 10.1016/S0306-4522(99)00582-5. 10683565

[ref32] Fasano C.; Miolan J. P.; Niel J. P. (2003) Modulation by C2 Ceramide of the Nicotinic Transmission within the Coeliac Ganglion in the Rabbit. Neuroscience 116 (3), 753–759. 10.1016/S0306-4522(02)00760-1. 12573717

[ref33] Zhang Y. H.; Vasko M. R.; Nicol G. D. (2002) Ceramide, a Putative Second Messenger for Nerve Growth Factor, Modulates the TTX-resistant Na^+2^ + Current and Delayed Rectifier K^+2^ + Current in Rat Sensory Neurons. J. Physiol. 544 (2), 385–402. 10.1113/jphysiol.2002.024265. 12381813 PMC2290585

[ref34] Dobrowsky R. T.; Werner M. H.; Castellino A. M.; Chao M. V.; Hannun Y. A. (1994) Activation of the Sphingomyelin Cycle through the Low-Affinity Neurotrophin Receptor. Science 265 (5178), 1596–1599. 10.1126/science.8079174. 8079174

[ref35] Fasano C.; Tercé F.; Niel J.-P.; Nguyen H. T. T.; Hiol A.; Bertrand-Michel J.; Mallet N.; Collet X.; Miolan J.-P. (2007) Neuronal Conduction of Excitation without Action Potentials Based on Ceramide Production. PLoS One 2 (7), e612 10.1371/journal.pone.0000612. 17637828 PMC1906860

[ref36] Kim H.-Y.; Huang B. X.; Spector A. A. (2014) Phosphatidylserine in the Brain: Metabolism and Function. Prog. Lipid Res. 56, 1–18. 10.1016/j.plipres.2014.06.002. 24992464 PMC4258547

[ref37] Baudry M.; Massicotte G.; Hauge S. (1991) Phosphatidylserine Increases the Affinity of the AMPA/Quisqualate Receptor in Rat Brain Membranes. Behav. Neural Biol. 55 (2), 137–140. 10.1016/0163-1047(91)80134-Z. 1647760

[ref38] Murray J.; Cuccia L.; Ianoul A.; Cheetham J. J.; Johnston L. J. (2004) Imaging the Selective Binding of Synapsin to Anionic Membrane Domains. ChemBioChem 5 (11), 1489–1494. 10.1002/cbic.200400097. 15481031

[ref39] Allen J. A.; Halverson-Tamboli R. A.; Rasenick M. M. (2007) Lipid Raft Microdomains and Neurotransmitter Signalling. Nat. Rev. Neurosci. 8 (2), 128–140. 10.1038/nrn2059. 17195035

[ref40] Bader Lange M. L.; Cenini G.; Piroddi M.; Mohmmad Abdul H.; Sultana R.; Galli F.; Memo M.; Butterfield D. A. (2008) Loss of Phospholipid Asymmetry and Elevated Brain Apoptotic Protein Levels in Subjects with Amnestic Mild Cognitive Impairment and Alzheimer Disease. Neurobiol. Dis. 29 (3), 456–464. 10.1016/j.nbd.2007.11.004. 18077176 PMC2292396

[ref41] Drago F.; Canonico P. L.; Scapagnini U. (1981) Behavioral Effects of Phosphatidylserine in Aged Rats. Neurobiol. Aging 2 (3), 209–213. 10.1016/0197-4580(81)90023-3. 7312099

[ref42] Glade M. J.; Smith K. (2015) Phosphatidylserine and the Human Brain. Nutrition 31 (6), 781–786. 10.1016/j.nut.2014.10.014. 25933483

[ref43] Cohen S. A.; Müller W. E. (1992) Age-Related Alterations of NMDA-Receptor Properties in the Mouse Forebrain: Partial Restoration by Chronic Phosphatidylserine Treatment. Brain Res. 584 (1–2), 174–180. 10.1016/0006-8993(92)90892-D. 1355390

[ref44] Rolandi R.; Robello M.; Mao C.; Mainardi P.; Besio G. (1990) Adsorption of γ-Aminobutyric Acid to Phosphatidylserine Membranes. Cell Biophys. 16 (1–2), 71 10.1007/BF02989693. 1691686

[ref45] Chweh A. Y.; Leslie S. W. (1982) Phosphatidylserine Enhancement of [3H]Γ-Aminobutyric Acid Uptake by Rat Whole Brain Synaptosomes. J. Neurochem. 38 (3), 691–695. 10.1111/j.1471-4159.1982.tb08687.x. 7057188

[ref46] De Medio G. E.; Trovarelli G.; Hamberger A.; Porcellati G. (1980) Synaptosomal Phospholipid Pool in Rabbit Brain and Its Effect on GABA Uptake. Neurochem. Res. 5 (2), 171–179. 10.1007/BF00964330. 6767992

[ref47] Tanguy E.; Wang Q.; Moine H.; Vitale N. (2019) Phosphatidic Acid: From Pleiotropic Functions to Neuronal Pathology. Front. Cell. Neurosci. 13 (January), 1–8. 10.3389/fncel.2019.00002. 30728767 PMC6351798

[ref48] Tracey T. J.; Steyn F. J.; Wolvetang E. J.; Ngo S. T. (2018) Neuronal Lipid Metabolism: Multiple Pathways Driving Functional Outcomes in Health and Disease. Front. Mol. Neurosci. 11, 10 10.3389/fnmol.2018.00010. 29410613 PMC5787076

[ref49] Daniel M. (2017) Raben and Casey N. Barber. Phosphatidic Acid and Neurotransmission Daniel. Adv. Biol. Regul. 63 (1), 15–21. 10.1016/j.jbior.2016.09.004. 27671966 PMC5292084

[ref50] Burger K. N.; Demel R. A.; Schmid S. L.; de Kruijff B. (2000) Dynamin Is Membrane-Active: Lipid Insertion Is Induced by Phosphoinositides and Phosphatidic Acid. Biochemistry 39 (40), 12485–12493. 10.1021/bi000971r. 11015230

[ref51] Truckenbrodt S.; Viplav A.; Jähne S.; Vogts A.; Denker A.; Wildhagen H.; Fornasiero E. F.; Rizzoli S. O. (2018) Newly Produced Synaptic Vesicle Proteins Are Preferentially Used in Synaptic Transmission. EMBO J. 37 (15), e98044 10.15252/embj.201798044. 29950309 PMC6068464

[ref52] Frisz J. F.; Lou K.; Klitzing H. A.; Hanafin W. P.; Lizunov V.; Wilson R. L.; Carpenter K. J.; Kim R.; Hutcheon I. D.; Zimmerberg J.; et al. (2013) Direct Chemical Evidence for Sphingolipid Domains in the Plasma Membranes of Fibroblasts. Proc. Natl. Acad. Sci. U. S. A. 110 (8), E613–E622. 10.1073/pnas.1216585110. 23359681 PMC3581929

[ref53] Sud M.; Fahy E.; Cotter D.; Brown A.; Dennis E. A.; Glass C. K.; Merrill A. H.; Murphy R. C.; Raetz C. R. H.; Russell D. W.; et al. (2007) LMSD: LIPID MAPS Structure Database. Nucleic Acids Res. 35 (SUPPL. 1), 527–532. 10.1093/nar/gkl838. PMC1669719 17098933

