# Supplementary material for: Secondary Ion Mass Spectrometry Imaging Reveals Changes in the Lipid Structure of the Plasma Membranes of Hippocampal Neurons following Drugs Affecting Neuronal Activity
Source: ACS Chem Neurosci. 2021 Apr 26;12(9):1542–51. doi: 10.1021/acschemneuro.1c00031 (PMC8154318; doi:10.1021/acschemneuro.1c00031)

Supporting Information

Secondary Ion Mass Spectrometry Imaging Reveals Changes in The Lipid Structure of the Plasma Membranes of Hippocampal Neurons Following Drugs Affecting Neuronal Activity

Paola Agüi-Gonzalez[#,¶], Bao Guobin[†], Maria Angela Gomes de Castro[#], Silvio O. Rizzoli[#,¶], and Nhu T.N. Phan[#,¶,§,*]

[#]Department of Neuro- and Sensory Physiology, University Medical Center Göttingen, Göttingen 37073, Germany
[¶]Center for Biostructural Imaging of Neurodegeneration, University Medical Center Göttingen, Göttingen 37075, Germany
[†]Department of Pharmacology and Toxicology, University Medical Center Göttingen, Göttingen 37075, Germany
[§]Department of Chemistry and Molecular Biology, University of Gothenburg, Gothenburg 41296, Sweden

Corresponding Author
Nhu T.N. Phan - Email: thi.phan@med.uni-goettingen.de/ nhu.phan@chem.gu.se.

Table S1. Peak composition of the most dominant ICs distributing at the plasma membrane of the cell body and neurites obtained by ICA

Postive	IC index No.	m/z										
Neurites	2	86,10	508,69	554,67	166,10	636,59	512,69					
	3	298,08	86,10	433,23	378,47	577,54	562,58	496,36	660,64			
	4	378,47	710,60	418,55	432,40	524,41	470,51	654,58				
	5	86,10	478,35	528,70	510,74	142,04	496,36					
	6	508,69	507,67	427,18	184,07	530,70	496,36					
	7	440,28	582,78	636,59	385,34	124,04						
	9	86,10	622,61	561,74	492,39	504,82	580,81	456,82				
	10	86,10	508,69	510,74	428,43	511,66						
	11	462,75	514,74	478,35	496,36	124,04						
Cell Body	36	184,07	86,10	433,23	577,54							
	37	440,28	184,07	337,25	224,10	649,62	478,35					
	39	86,10	509,61	166,04								
	40	414,81	732,58									
	41	124,04	654,65	552,50	492,39	142,04	385,34	184,07	446,31	606,66		
	43	404,80	184,07	508,69	339,27	367,31	580,63					
Negative												
Neurites	1	223,06	140,05	340,39								
	2	241,10	408,32	523,49	199,20							
	3	452,45	534,68	394,02	496,37	126,06						
	5	199,20	255,22	313,14	460,39	277,24						
	6	283,23	255,22	311,30	256,27	861,81	833,79	227,22	325,38	408,32	279,23	281,28
	7	180,08	299,02	126,06	406,45	241,07	227,22	444,38	126,06			
	8	166,10	313,06	320,17	507,67	255,22	337,25	446,20	502,88	470,51		
	9	241,10	227,22	283,23	303,27	140,05	301,14	464,30	255,40	180,08	413,38	
	10	241,07	140,05	277,24	460,39	544,37						
Cell Body	49	281,28	241,03	316,04								

Table S2. Tentative peak assignment in positive and negative SIMS mode.

**Positive mode**				
**Tentative Assignment**	**Formula**	**Detected mass**	**Theoretical mass**	**Delta**
**Ceramide**				
Cer(36:1;O2)	$C_{36}H_{72}NO_3$	566.59	566.55	68.50
Cer(36:1;O3)	$C_{36}H_{72}NO_4$	582.59	582.55	70.20
Cer(36:0;O3)+Na	$C_{36}H_{73}NO_4Na$	606.55	606.54	2.14
CerP(34:1)+Na	$C_{34}H_{68}NO_6PNa$	640.47	640.47	9.10
CerP(34:0;O2)+K	$C_{34}H_{70}NO_6PK$	658.49	658.46	42.89
GalCer(32:1)+K	$C_{38}H_{73}NO_8K$	710.51	710.50	10.84
**Phosphatidic Acid**				
PA(12:0)+K	$C_{15}H_{31}O_7PK$	393.16	393.14	31.95
PA(36:2)+Na	$C_{39}H_{73}O_8PNa$	723.48	723.49	22.18
PA(39:1)+K	$C_{42}H_{81}O_8PK$	783.54	783.53	12.02
PA(41:2)+K	$C_{44}H_{83}O_8PK$	809.56	809.55	15.77
PA(44:8)+Na	$C_{47}H_{77}O_8PNa$	823.53	823.53	8.08
**Phosphatidylcholine**				
PC fragment	$C_5H_{13}PNO_3$	166.06	166.06	0.41
PC head group	$C_5H_{15}PNO_4$	184.07	184.07	0.73
PC fragment	$C_8H_{19}NPO_4$	224.12	224.11	43.89
PC(28:2)	$C_{36}H_{70}NO_8P$	674.49	674.48	16.23
PC(28:1)	$C_{36}H_{72}NO_8P$	676.49	676.49	3.39
PC(30:0)	$C_{38}H_{76}NO_8P$	706.51	706.53	28.31
PC(32:1)+Na	$C_{40}H_{78}NO_8PNa$	754.52	754.54	20.21
PC(34:2)+Na	$C_{42}H_{80}NO_8PNa$	780.58	780.55	36.00
PC(34:1)+Na	$C_{42}H_{82}NO_8PNa$	782.58	782.57	18.48
**Phosphatidylglycerol**				
PG(28:0;O)+Na	$C_{34}H_{69}O_9PNa$	675.47	675.46	16.78
PG(25:1;O)+K	$C_{31}H_{59}O_{11}PK$	677.34	677.34	4.71
PG(31:1;O)	$C_{37}H_{74}O_9P$	693.50	693.51	8.68
PG(30:0)	$C_{36}H_{72}O_{10}P$	695.46	695.49	32.04
PG(41:6)	$C_{47}H_{81}O_{10}PK$	875.54	875.52	22.37
**Phosphatidylserine**				
PS(36:6)+K	$C_{42}H_{70}NO_{10}PK$	818.46	818.44	27.59
PS(40:6)+K	$C_{46}H_{78}NO_{10}PK$	874.50	874.50	0.02
PS(43:6)+Na	$C_{49}H_{84}NO_{10}PNa$	900.55	900.57	22.23
**Diacylglycerol**				
DG(31:3)+Na	$C_{34}H_{60}O_5Na$	571.44	571.43	12.56
DG(33:3)	$C_{36}H_{65}O_5$	577.50	577.48	23.94

DG(35:6)	$C_{38}H_{63}O_5$	599.47	599.47	4.13
DG(37:7)	$C_{40}H_{65}O_5$	625.48	625.48	0.28
DG(37:6)	$C_{40}H_{67}O_5$	627.51	627.50	22.59
DG(40:1)	$C_{43}H_{83}O_5$	679.64	679.62	23.52
DG(43:6)+K	$C_{46}H_{78}O_5K$	749.55	749.55	1.87

Negative mode

Tentative assignment	Formula	Detected mass	Theoretical mass	Delta
**Ceramides**				
Cer(36:1)	$C_{36}H_{70}NO_3$	564.56	564.54	40.85
Cer(38:1;O)	$C_{38}H_{74}NO_2$	576.58	576.57	17.51
Cer(36:0;O3)	$C_{36}H_{72}NO_4$	582.56	582.55	31.40
Cer(39:2;O2)	$C_{39}H_{74}NO_3$	604.57	604.57	1.57
Cer(40:1;O2)	$C_{40}H_{78}N_{O3}$	620.57	620.60	49.84
GlcCer(36:2;O2)	$C_{42}H_{78}NO_8$	724.56	724.57	15.57
**Fatty Acids**				
FA(16:1)	$C_{16}H_{29}O_2$	253.21	253.22	7.71
FA(20:5)	$C_{20}H_{31}O_2$	303.22	303.23	33.02
FA(38:3)	$C_{38}H_{69}O_2$	557.55	557.53	30.75
**Phosphatidic Acid**				
PA(34:4;O)	$C_{37}H_{66}O_7P$	653.51	653.45	90.62
PA(34:3;O)	$C_{37}H_{68}O_7P$	655.49	655.47	27.23
PA(34:0;O)	$C_{37}H_{74}O_7P$	661.52	661.52	5.28
PA(35:3;O)	$C_{38}H_{70}O_7P$	669.49	669.49	3.20
PA(35:2)	$C_{38}H_{70}O_8P$	685.50	685.48	28.38
PA(36:1)	$C_{39}H_{74}O_8P$	701.53	701.51	24.49
PA(36:0)	$C_{39}H_{76}O_8P$	703.54	703.53	20.12
PA(38:5;O)	$C_{41}H_{72}O_7P$	707.52	707.50	23.80
PA(38:3;O)	$C_{41}H_{76}O_7P$	711.54	711.53	5.23
PA(38:5)	$C_{41}H_{70}O_8P$	721.49	721.48	17.10
PA(38:4)	$C_{41}H_{72}O_8P$	723.51	723.50	22.88
PA(39:1)	$C_{42}H_{76}O_8P$	739.52	739.53	10.69
PA(39:2)	$C_{42}H_{78}O_8P$	741.54	741.54	5.46
**Phosphatidylethanolamine**				
PE-Cer(32:1;O2)	$C_{34}H_{68}N_2O_6P$	631.52	631.48	64.49
**Phosphatidylinositol**				
PI-Cer(46:0;O3)	$C_{52}H_{103}NO_{12}P$	964.70	964.72	24.46
**Phosphatidylserine**				
PS(39:2)	$C_{45}H_{83}NO_{10}P$	828.55	828.58	36.63
PS(43:4)	$C_{49}H_{88}NO_{10}P$	880.61	880.61	7.12
**Triacylglycerol**				
TG(37:1)	$C_{40}H_{73}O_6$	649.54	649.54	5.07
TG(37:0)	$C_{40}H_{75}O_6$	651.54	651.56	30.49
TG(53:8)	$C_{56}H_{91}O_6$	859.64	859.68	44.19
TG(53:7)	$C_{56}H_{93}O_6$	861.71	861.70	10.35
TG(53:6)	$C_{56}H_{95}O_6$	863.71	863.71	2.64

TG(54:3)	$C_{57}H_{103}O_6$	883.78	883.78	3.73
TG(54:2)	$C_{57}H_{105}O_6$	885.79	885.79	2.27
TG(54:1)	$C_{57}H_{107}O_6$	887.80	887.81	12.01
TG(55:7)	$C_{58}H_{97}O_6$	889.70	889.73	27.57
TG(60:11)	$C_{63}H_{99}O_6$	951.72	951.74	26.69

Table S3. Relative change of membrane lipid compositions in hippocampal neurons following drug treatments (TTX, BIC). The percentages represent the relative number of lipid compounds changed by the drug treatments.

LIPID SUBCLASS	Body TTX ↑	Body TTX ↓	Neur TTX ↑	Neur TTX ↓	Body BIC ↑	Body BIC ↓	Neur BIC ↑	Neur BIC ↓
Cer	83%	0%	83%	8%	0%	0%	0%	0%
FA	33%	0%	33%	0%	67%	0%	100%	0%
PA	67%	0%	22%	0%	44%	0%	11%	0%
PC	22%	33%	44%	0%	33%	44%	11%	0%
PG	20%	0%	40%	0%	40%	0%	40%	0%
PS	0%	20%	0%	60%	40%	0%	20%	0%
TG	60%	0%	40%	0%	80%	0%	40%	0%

Figure S1. Comparison of CCD values between the control and TTX treatment in the cell body in positive ion mode

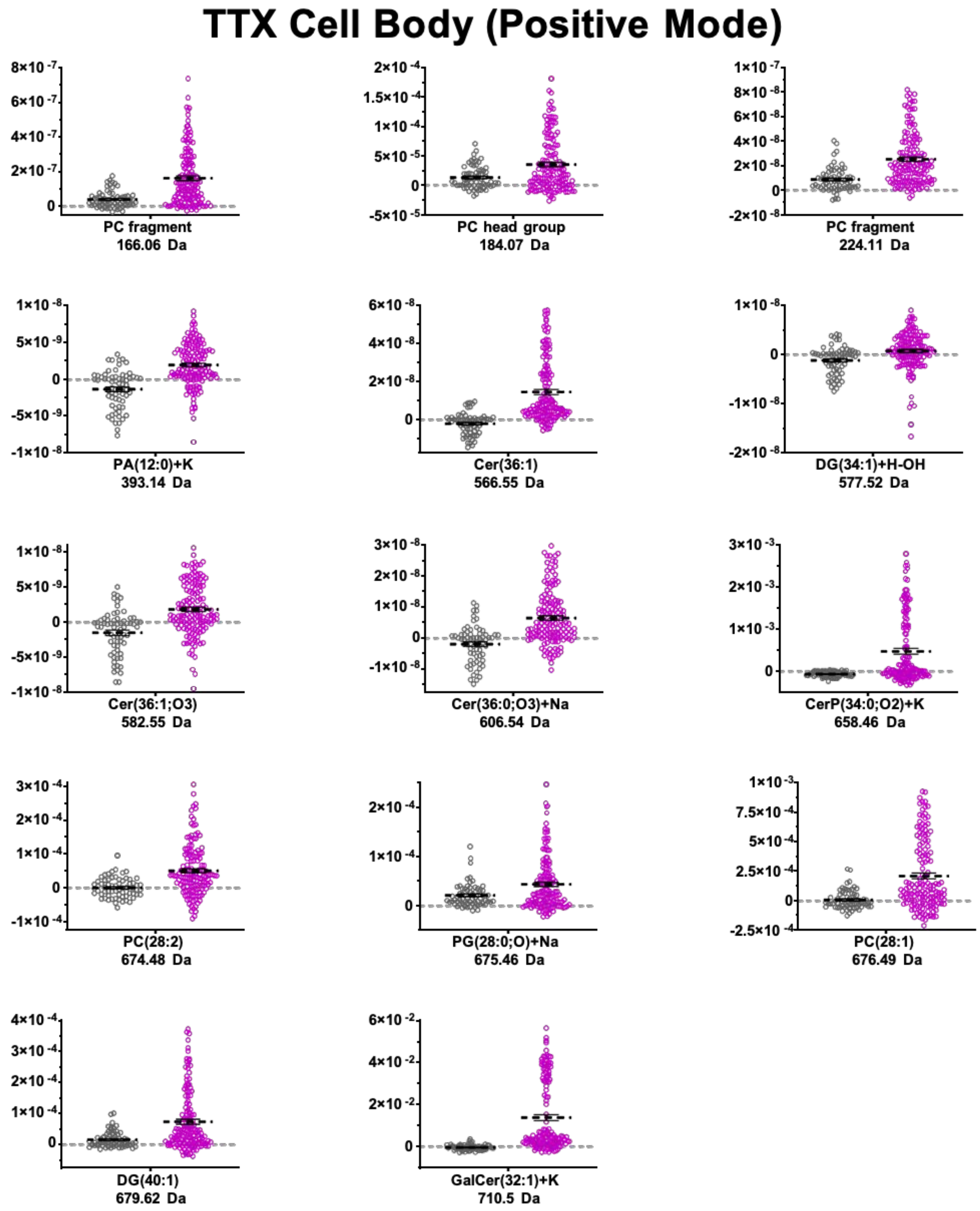

Figure S2. **Comparison of CCD values between the control and TTX treatment in the neurites in positive ion mode**

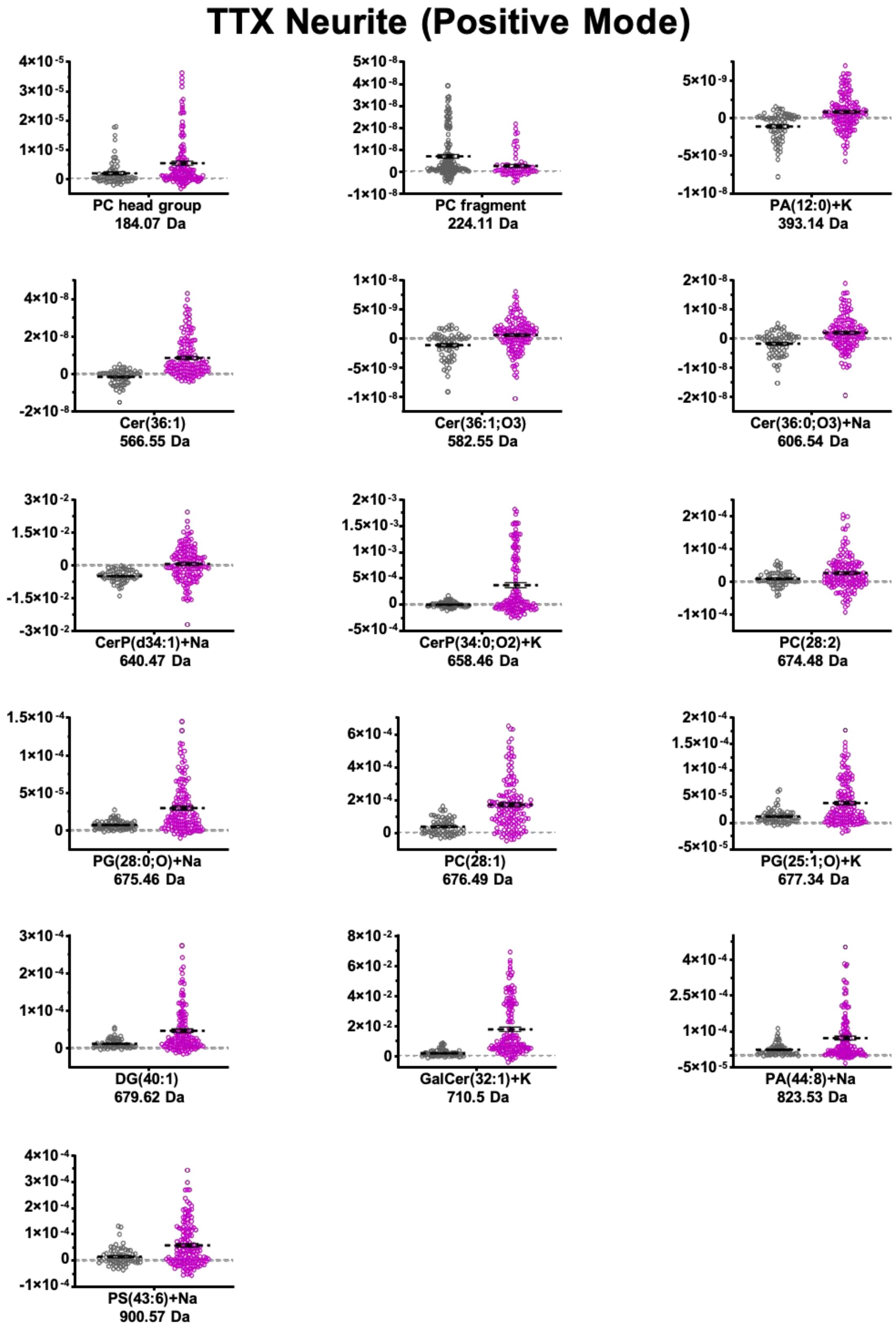

Figure S3. Comparison of CCD values between the control and BIC treatment in the cell body in positive ion mode

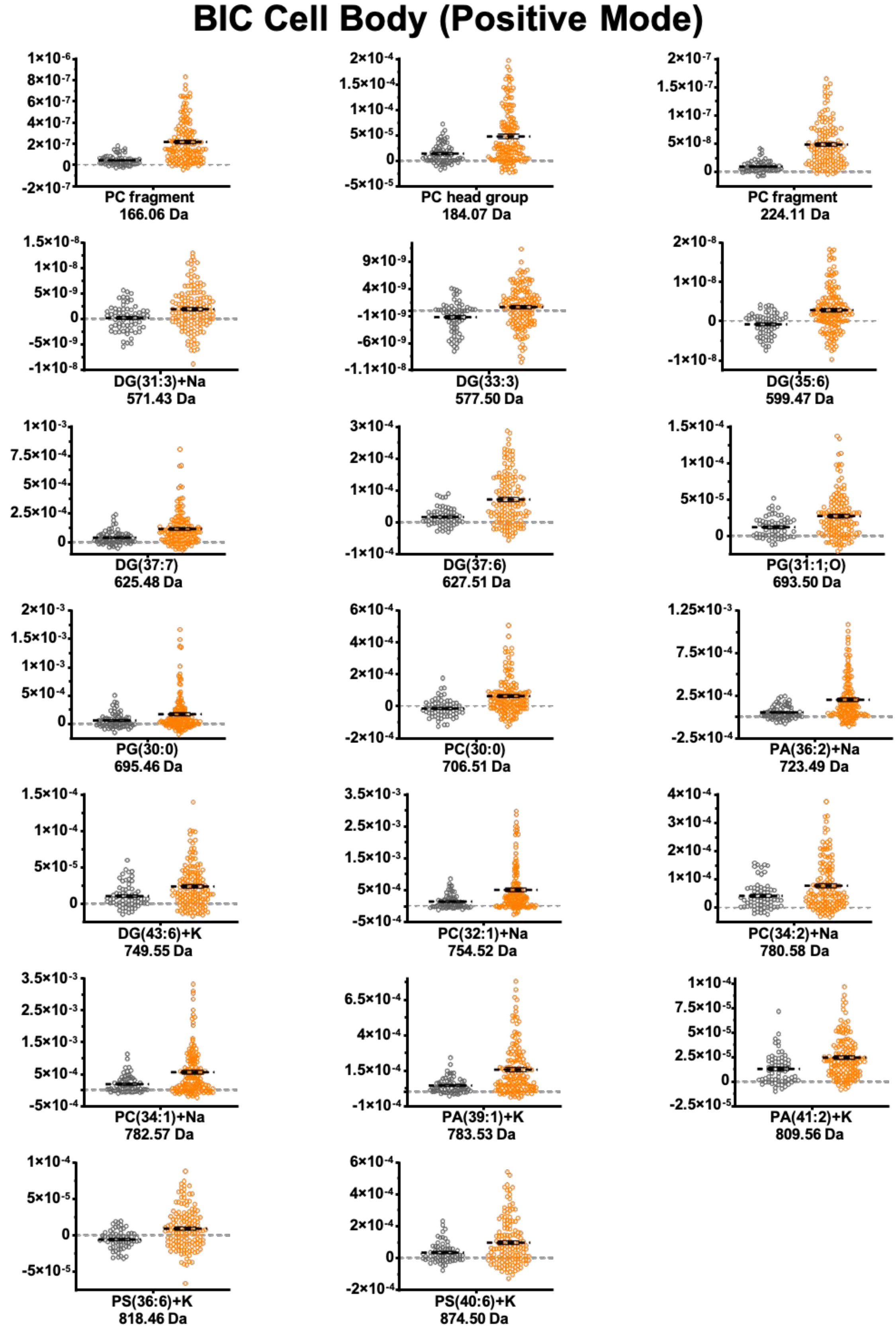

Figure S4. Comparison of CCD values between the control and BIC treatment in the neurites in positive ion mode

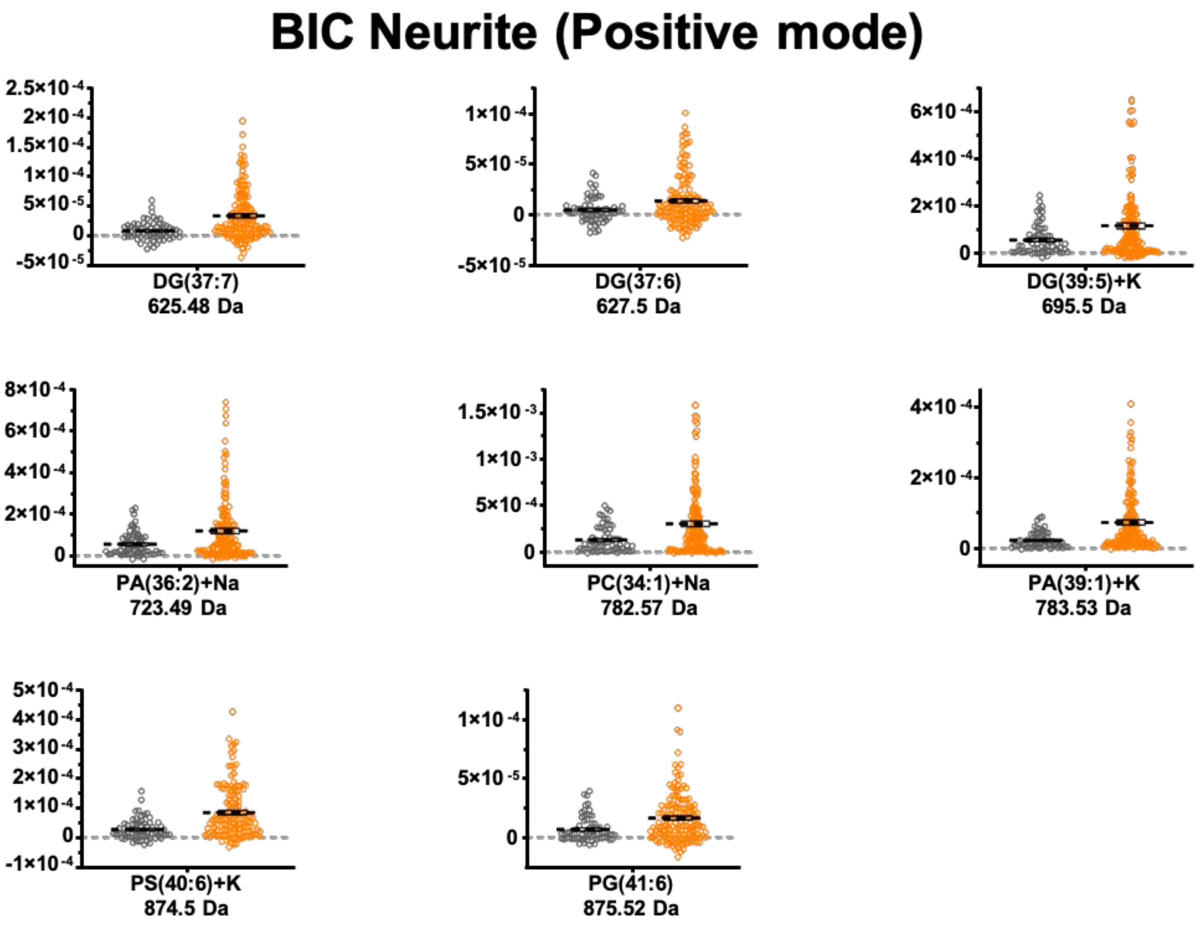

Figure S5. Comparison of CCD values between the control and TTX treatment in the cell body in negative ion mode

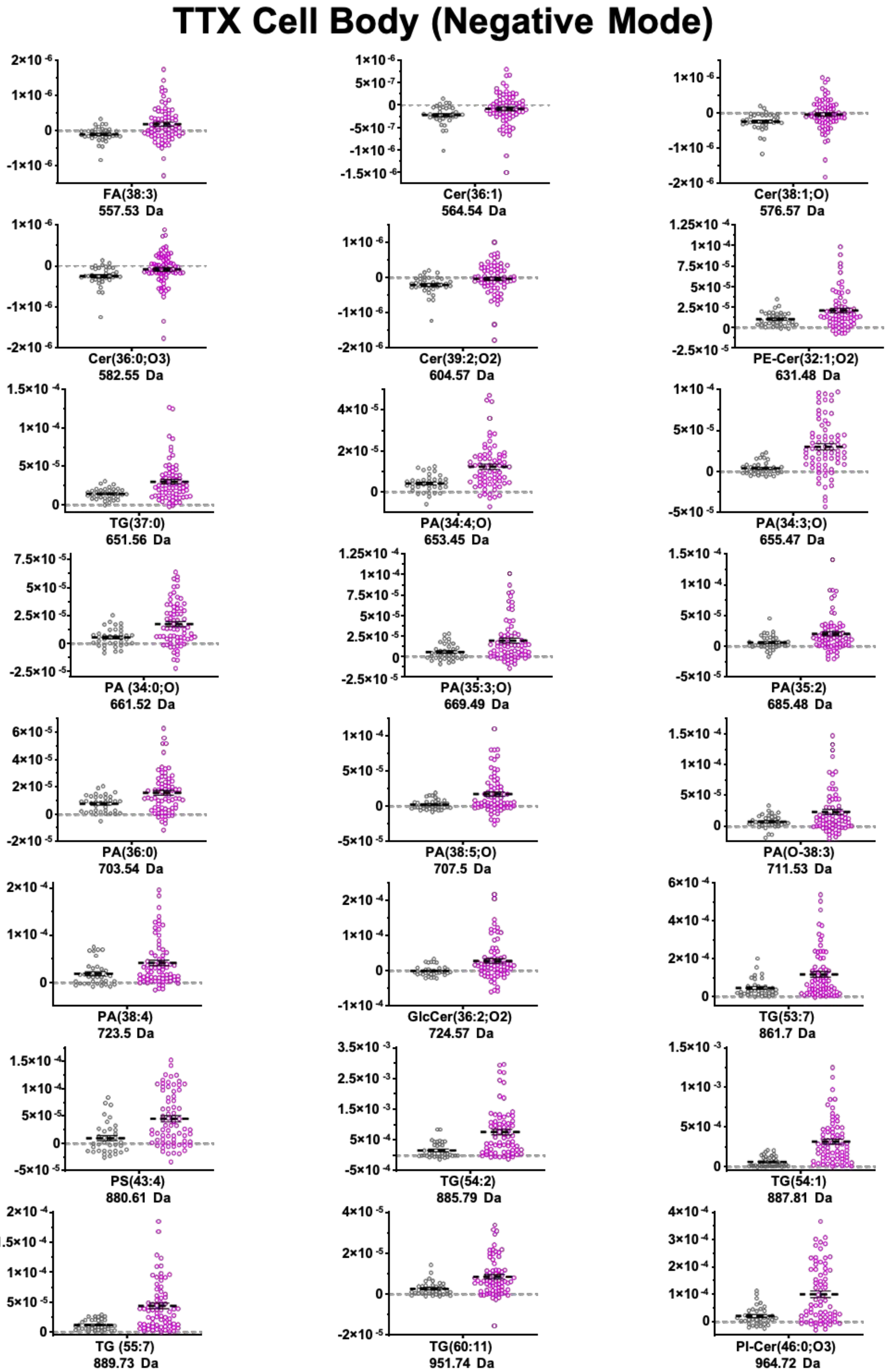

Figure S6. **Comparison of CCD values between the control and TTX treatment in the neurites in negative ion mode**

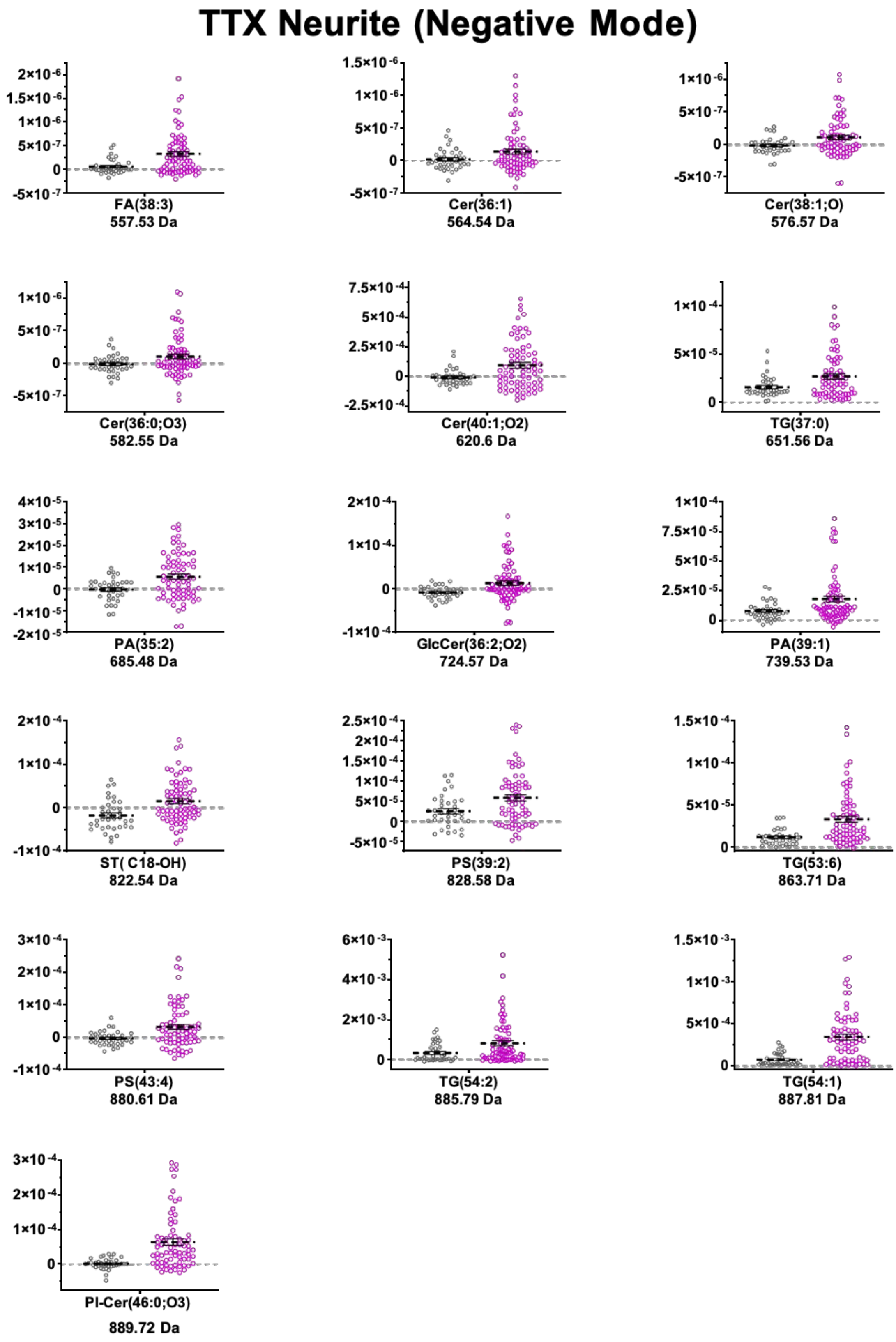

Figure S7. **Comparison of CCD values between the control and BIC treatment in the cell body in negative ion mode**

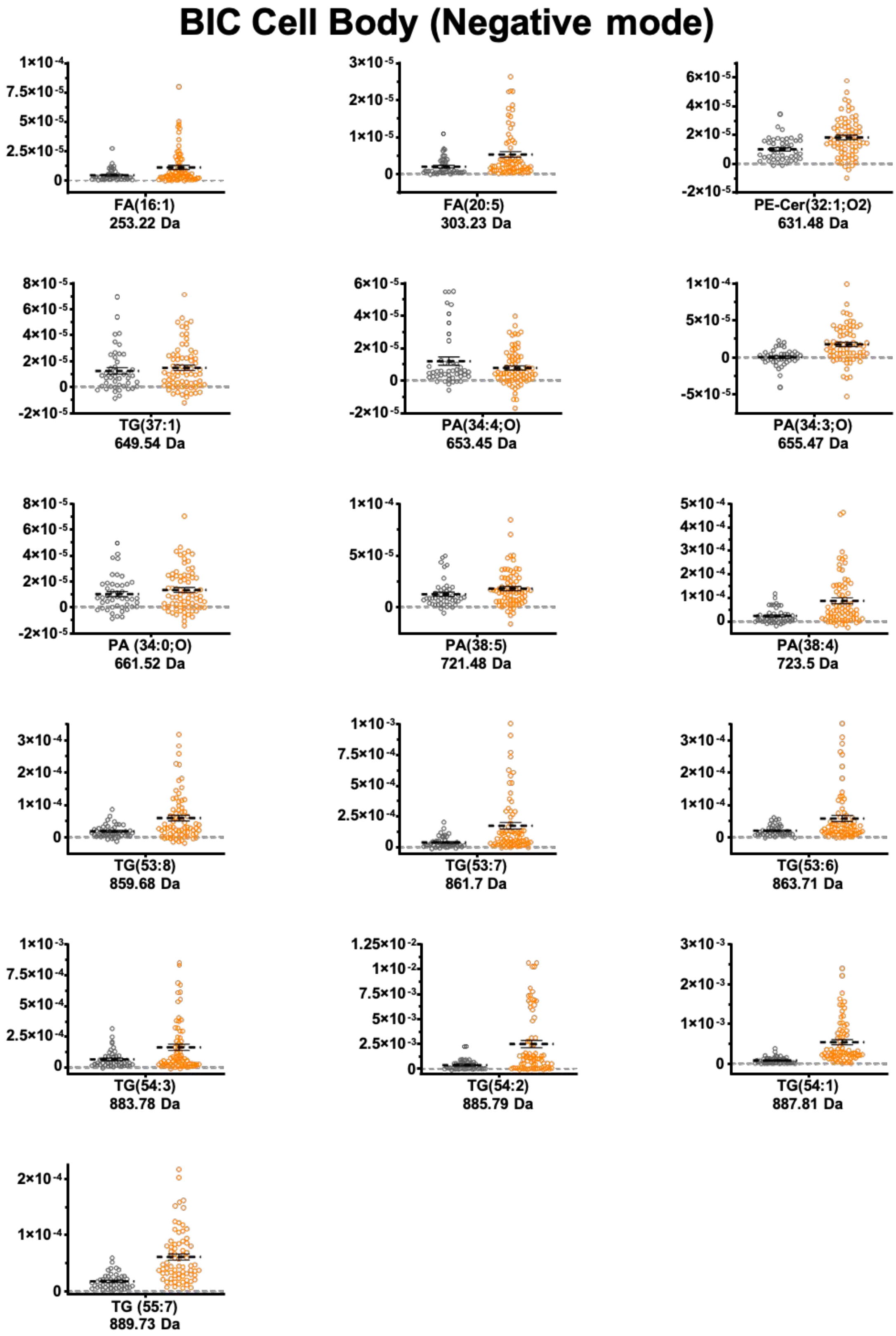

Figure S8. **Comparison of CCD values between the control and BIC treatment in the neurites in negative ion mode**

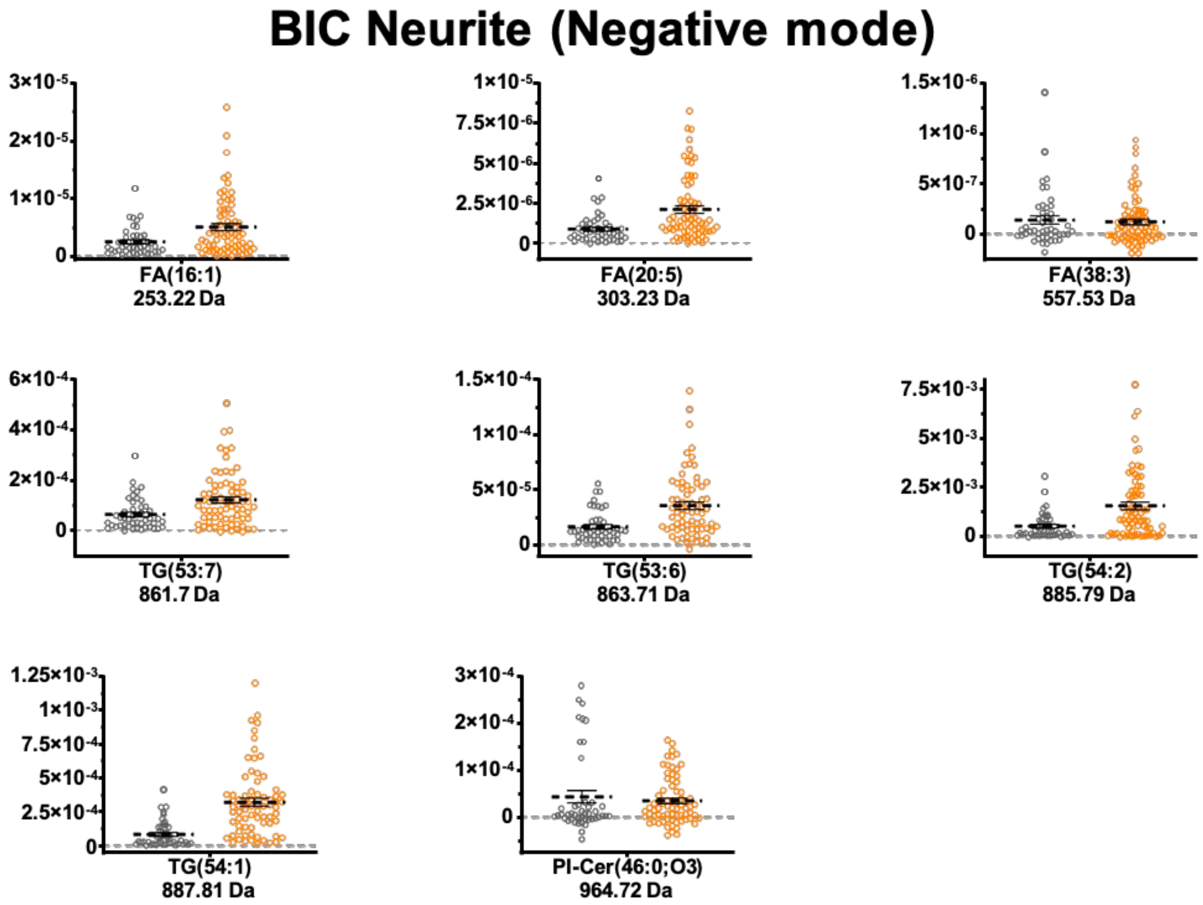

Supplement: Supplementary file 1 — cn1c00031_si_001.pdf [file cn1c00031_si_001.pdf]
